# Psyllium and Glucomannan as Viscous Fiber Modulators of the Gut–Microbiome–Incretin Axis: Molecular Links to Metabolic Inflammation, Barrier Function, and GLP-1 Receptor Agonist Therapy

**DOI:** 10.3390/ijms27146287

**Published:** 2026-07-15

**Authors:** Yonghyun Yoon, Jihyo Hwang, Chan-Mo Yang, Myunghoon Moon, Jong-Jin Lee, King Hei Stanley Lam, Jeimylo C. de Castro, Teinny Suryadi, Anwar Suhaimi, Jonghyeok Lee

**Affiliations:** 1 Department of Orthopaedic Surgery, Gangnam Sacred Heart Hospital, Hallym University College of Medicine, 1 Singil-ro, Yeongdeungpo-gu, Seoul 07441, Republic of Korea; mgyyh00@gmail.com (Y.Y.);; 2 Incheon Terminal Orthopedic Surgery Clinic, Inha-ro 489beon-gil, Namdong-gu, Incheon 21574, Republic of Korea; 3 International Academy of Regenerative Medicine, Inha-ro 489beon-gil, Namdong-gu, Incheon 21574, Republic of Korea; 4 The Board of Clinical Research, International Academy of Musculoskeletal Medicine, Kowloon, Hong Kong, China; 5 Department of Psychiatry, School of Medicine, Wonkwang University, Iksan 54538, Republic of Korea; 6 Naeuram Clinic, Seoul 05363, Republic of Korea; 7 Ceramique Clinic, Seoul 06123, Republic of Korea; 8 Faculty of Medicine, The University of Hong Kong, Pokfulam, Hong Kong, China; 9 Faculty of Medicine, The Chinese University of Hong Kong, New Sha Tin, New Territories, Hong Kong, China; 10 SMARTMD Center for Non-Surgical Pain Interventions, Makati 1205, Philippines; jeidec@yahoo.com.ph; 11 Research Department, Adventist University of the Philippines, Silang 4118, Philippines; 12 Department of Physical Medicine and Rehabilitation, Medistra Hospital, South Jakarta 12950, Indonesia; 13 Department of Physical Medicine and Rehabilitation, Synergy Clinic, West Jakarta 11510, Indonesia; 14 Department of Rehabilitation Medicine, Universiti Malaya, Kuala Lumpur 50603, Malaysia; 15 Bareun Neurosurgery Clinic, 39, Daenong-ro, Heungdeok-gu, Cheongju 28402, Republic of Korea

**Keywords:** dietary fiber, psyllium, glucomannan, gut microbiota, short-chain fatty acids, GLP-1 receptor agonists, intestinal barrier, type 2 diabetes, enteroendocrine signaling, cardiometabolic risk

## Abstract

Dietary fiber is an under-consumed nutritional substrate that supports gut microbial metabolism, intestinal barrier integrity, enteroendocrine signaling, and cardiometabolic regulation. Among dietary fibers, psyllium and glucomannan are clinically accessible viscous, gel-forming soluble fibers with distinct but complementary physicochemical profiles. This narrative review examines how these fibers act through luminal viscosity, nutrient diffusion, bile acid and cholesterol handling, short-chain fatty acid production, epithelial barrier support, enteroendocrine L-cell signaling, and low-grade metabolic inflammation. Psyllium has the strongest clinical support for stool normalization, glycemic modulation, and LDL cholesterol reduction, whereas glucomannan provides marked viscosity and water-holding capacity that may support satiety, lipid modulation, and weight-management strategies when appropriately hydrated and tolerated. We also discuss the emerging nutritional context of glucagon-like peptide-1 receptor agonist therapy, in which appetite suppression, reduced meal volume, delayed gastrointestinal transit, and constipation may reduce dietary fiber intake and fermentable substrate delivery to the colon. The pain- and mood-related implications are framed as hypothesis-generating extensions, because direct clinical evidence that psyllium or glucomannan improves these outcomes remains limited. A psyllium-centered, selectively glucomannan-supported strategy may help close the fiber gap and support bowel function, microbial metabolite signaling, and cardiometabolic stability during modern metabolic and weight-loss therapies.

## 1. Introduction

Dietary fiber is a biologically active nutritional substrate that has been progressively depleted from modern diets [1]. For most of human evolutionary history, plant-derived fibers from wild plants, tubers, fruits, seeds, and other unrefined foods provided a continuous and diverse substrate for the intestinal microbiota. In contrast, contemporary dietary patterns characterized by refined carbohydrates, ultra-processed foods, low plant diversity, and reduced whole-food intake have markedly decreased daily fiber exposure [2]. This shift has occurred over a period too brief for meaningful host-microbial adaptation and has created a mismatch between modern dietary habits and the metabolic ecology of the human gut [3].

Current dietary guidance commonly recommends approximately 14 g of total fiber per 1000 kcal, corresponding to about 25 g/day for adult women and 38 g/day for adult men, with European recommendations generally falling within a similar range of approximately 25–35 g/day [4,5]. These values should be interpreted as population-level intake targets and practical benchmarks for identifying the “fiber gap,” rather than as validated therapeutic doses that uniformly reverse dysbiosis, impaired SCFA production, increased gut permeability, endotoxemia, insulin resistance, dyslipidemia, endothelial dysfunction, or chronic inflammatory activation.

Traditionally, dietary fiber has been discussed mainly in relation to bowel regularity and constipation [6]. However, this view substantially underestimates its physiological relevance. Fermentable and viscous fibers influence nutrient absorption, bile acid metabolism, intestinal transit, gut barrier integrity, microbial diversity, short-chain fatty acid (SCFA) production, enteroendocrine signaling, and systemic immune regulation [7]. Through these pathways, dietary fiber can affect metabolic health far beyond the gastrointestinal tract. Insufficient fiber intake may contribute to dysbiosis, impaired SCFA production, increased gut permeability, low-grade endotoxemia, insulin resistance, dyslipidemia, endothelial dysfunction, and chronic inflammatory activation [8]. These mechanisms overlap with the pathophysiology of metabolic syndrome, type 2 diabetes, atherosclerotic cardiovascular disease, and several chronic inflammatory conditions [9].

**Figure 1:**
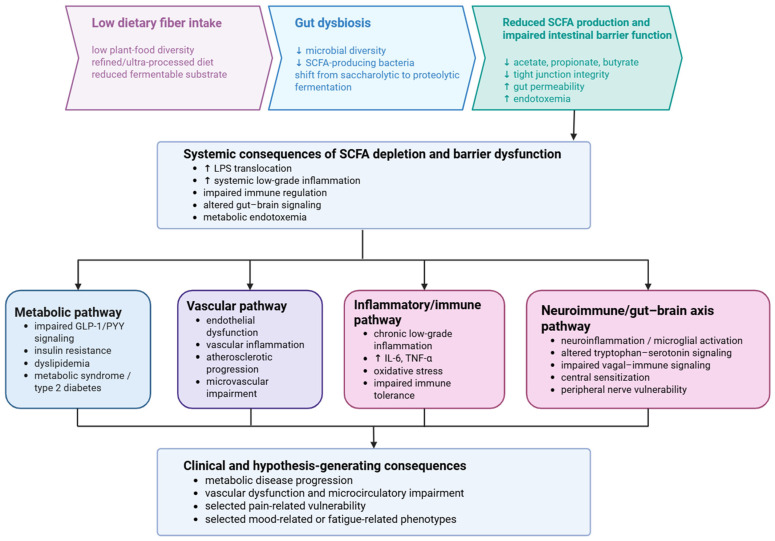
Low-fiber intake and the gut–microbiome–metabolic–vascular–neuroimmune axis. Low dietary fiber intake may reduce fermentable substrate availability, impair short-chain fatty acid production, weaken epithelial barrier integrity, and increase gut-derived inflammatory signaling. These changes may contribute to insulin resistance, dyslipidemia, endothelial dysfunction, chronic low-grade inflammation, and neuroimmune activation. The figure summarizes the proposed molecular and physiological links between inadequate fiber intake and metabolic, vascular, gastrointestinal, and selected pain-related phenotypes. Arrows indicate proposed directional relationships; upward and downward arrows indicate increases and decreases, respectively. SCFA, short-chain fatty acid; LPS, lipopolysaccharide.

At the cellular level, reduced fermentable substrate availability can decrease microbial production of acetate, propionate, and butyrate [10,11]. Butyrate serves as an important energy substrate for colonocytes and supports tight junction integrity, mucin-layer maintenance, and epithelial barrier function. When this barrier-supportive signaling is weakened, bacterial products such as lipopolysaccharide may more readily translocate across the intestinal mucosa and activate innate immune pathways, including TLR4- and NF-κB-related inflammatory signaling [8,12,13]. In parallel, reduced SCFA signaling through FFAR2/GPR43, FFAR3/GPR41, and GPR109A may impair enteroendocrine, immune, metabolic, and vascular homeostasis [10,14,15,16]. These pathways provide a molecular explanation for how insufficient fiber intake may contribute to low-grade endotoxemia, insulin resistance, dyslipidemia, endothelial dysfunction, and chronic inflammatory activation. These interconnected molecular and physiological pathways are summarized in Figure 1.

Among the heterogeneous group of dietary fibers, viscous gel-forming soluble fibers are especially important because their physicochemical properties directly influence nutrient diffusion, bile acid handling, and stool hydration. Psyllium, derived from Plantago ovata husk, and glucomannan, derived from konjac, are two representative fibers with distinct but complementary physicochemical properties [17]. Psyllium forms a hydrated gel within the intestinal lumen, modulating stool consistency, glucose diffusion, cholesterol absorption, and bile acid handling [18]. Glucomannan is a highly viscous, water-absorbing fiber that may contribute to satiety, delayed nutrient absorption, lipid modulation, and weight control [19]. Unlike non-viscous or poorly gel-forming fibers, these agents exert clinically meaningful effects through both mechanical and metabolic mechanisms [20]. Therefore, psyllium and glucomannan should not be viewed merely as laxatives or bulking agents, but as targeted nutritional interventions acting at the gut-metabolic interface. Because these fibers are not systemically absorbed as intact parent compounds, their clinical relevance is better understood through gastrointestinal physicochemical behavior, microbial fermentation, SCFA-mediated host signaling, and safety factors such as hydration, swallowing function, gastrointestinal motility, dose titration, and medication timing [10,21]. These structural, physicochemical, safety, and pharmacokinetic-like considerations are therefore addressed explicitly before the disease-specific and GLP-1 receptor agonist-related sections.

The metabolic implications of dietary fiber are especially relevant in patients with obesity, insulin resistance, type 2 diabetes, dyslipidemia, and metabolic syndrome [22]. These conditions are not isolated biochemical abnormalities, but systemic disorders involving altered nutrient handling, adipose tissue inflammation, endothelial dysfunction, oxidative stress, and impaired microvascular homeostasis. By attenuating postprandial glycemic excursions, improving lipid profiles, supporting satiety, and promoting SCFA-mediated anti-inflammatory signaling, viscous soluble fibers may address multiple components of cardiometabolic risk simultaneously [23]. This multidimensional effect is clinically important because metabolic syndrome and atherosclerosis share overlapping inflammatory and vascular mechanisms [24].

A further, more cautious implication concerns pain-related metabolic risk. Chronic pain phenotypes frequently coexist with obesity, insulin resistance, dyslipidemia, endothelial dysfunction, oxidative stress, and low-grade inflammation [25,26,27]. These systemic abnormalities may influence nociceptor sensitization, peripheral nerve vulnerability, microvascular perfusion, and neuroimmune activation. However, direct clinical evidence that psyllium or glucomannan improves pain outcomes remains limited [28]. Therefore, this review discusses pain-related phenotypes only as hypothesis-generating examples of shared metabolic-inflammatory pathways, not as established therapeutic indications for dietary fiber.

The gut–brain axis provides another hypothesis-generating context. Fiber-derived SCFAs may influence systemic immune tone, vagal signaling, blood–brain barrier integrity, microglial activity, hypothalamic–pituitary–adrenal axis regulation, and neurotransmitter-related pathways [29]. Nevertheless, most human evidence linking fiber intake to mood-related outcomes remains observational, and causality is uncertain [30]. Accordingly, dietary fiber should not be presented as a psychiatric treatment, but may be discussed as a supportive nutritional factor within the broader metabolic and neuroimmune environment.

The role of dietary fiber also requires reconsideration in the current era of glucagon-like peptide-1 receptor agonists (GLP-1 RAs) and dual GIP/GLP-1 receptor agonists [31]. These agents have transformed obesity and diabetes management by inducing substantial weight loss, reducing appetite, improving glycemic control, and altering gastrointestinal motility. However, appetite suppression, reduced total food intake, delayed gastric emptying, and constipation may unintentionally decrease the quantity and diversity of fermentable dietary substrates reaching the colon [32]. This reduction may have underappreciated consequences for microbial diversity, SCFA production, bowel function, endogenous enteroendocrine signaling, and maintenance of weight and glycemic benefits [33]. As weight regain after discontinuation of GLP-1-based pharmacotherapy remains a major clinical challenge, strategies that preserve dietary quality, fiber exposure, bowel function, and microbiome substrate availability are increasingly relevant [34]. This review therefore treats GLP-1 receptor agonist therapy as a nutritional context in which fiber sufficiency, bowel function, microbial substrate delivery, and cardiometabolic maintenance may require active clinical attention, rather than as evidence that fiber supplementation directly augments pharmacological incretin effects.

In this context, dietary fiber may be better considered a core nutritional substrate rather than an optional dietary component. Importantly, microbiome restoration should not be conceptualized solely as probiotic supplementation. Probiotics may have limited durability if the ecological substrate required for microbial engraftment and metabolic activity remains insufficient [35]. Fermentable dietary fibers provide the nutritional foundation for resident microbial communities, SCFA production, intestinal barrier maintenance, and host-microbial signaling. Accordingly, probiotics and synbiotics are best interpreted as adjunctive strategies whose durability may depend partly on adequate prebiotic substrate availability.

This narrative review focuses on psyllium and glucomannan as representative viscous, gel-forming fibers that modulate the gut–microbiome–incretin axis through luminal nutrient diffusion, bile acid and cholesterol handling, microbial fermentation, SCFA–GPCR signaling, epithelial barrier integrity, enteroendocrine function, metabolic inflammation, and vascular regulation. Pain- and mood-related outcomes are discussed only as secondary, hypothesis-generating extensions of these metabolic and neuroimmune pathways.

## 2. Narrative Review Approach

This article was designed as a narrative review rather than a systematic review or meta-analysis [36]. The aim was to synthesize mechanistic, translational, and clinical evidence linking viscous dietary fibers, particularly psyllium and glucomannan, to gut microbiome function, short-chain fatty acid (SCFA) signaling, incretin biology, metabolic inflammation, vascular dysfunction, and GLP-1 receptor agonist-associated gastrointestinal and nutritional effects, with pain- and mood-related outcomes considered only as secondary hypothesis-generating contexts.

Relevant literature was identified through searches of PubMed/MEDLINE, Scopus, Web of Science, and Google Scholar, supplemented by manual screening of reference lists from key reviews, clinical trials, mechanistic studies, and regulatory or guideline documents. Search terms included combinations of the following: “dietary fiber,” “psyllium,” “Plantago ovata,” “glucomannan,” “konjac,” “viscous fiber,” “soluble fiber,” “gut microbiota,” “short-chain fatty acids,” “SCFA,” “GLP-1,” “GLP-1 receptor agonist,” “incretin,” “metabolic syndrome,” “type 2 diabetes,” “dyslipidemia,” “atherosclerosis,” “inflammation,” “intestinal barrier,” “endotoxemia,” “probiotics,” “prebiotics,” “neuropathic pain,” “ischemic pain,” “adhesive capsulitis,” “neuroinflammation,” and “gut–brain axis.” Pain- and mood-related terms were included to identify mechanistic and translational context, not to establish dietary fiber as a direct therapeutic intervention for these outcomes.

Priority was given to peer-reviewed original studies, randomized controlled trials, meta-analyses, systematic reviews, mechanistic experimental studies, and authoritative regulatory or clinical guidance documents. Studies were selected if they were relevant to one or more of the following domains: physicochemical properties of dietary fibers; psyllium or glucomannan supplementation; glycemic, lipid, weight, or bowel outcomes; microbiome and SCFA-mediated mechanisms; enteroendocrine and GLP-1-related signaling; vascular inflammation and atherosclerosis; GLP-1 receptor agonist-associated gastrointestinal effects; and secondary, mechanistically linked pain- or neuroimmune-related contexts.

Because this was a narrative synthesis, no formal protocol registration, quantitative meta-analysis, or standardized risk-of-bias assessment was performed. The evidence was integrated conceptually to identify converging mechanisms, translational implications, and clinically relevant knowledge gaps. Particular caution was applied when discussing pain-related and mood-related outcomes, because direct interventional evidence for fiber supplementation in these domains remains limited. These topics were therefore framed as mechanistically plausible and hypothesis-generating rather than as established therapeutic indications.

## 3. Functional Classification of Dietary Fiber

Dietary fiber refers to a heterogeneous group of non-digestible carbohydrates and lignin that resist hydrolysis by human digestive enzymes in the small intestine and reach the colon either partially or completely intact [21,35]. Although dietary fibers are commonly categorized as soluble or insoluble, this binary classification is insufficient to explain their diverse physiological effects [6,21]. From a clinical and mechanistic perspective, dietary fibers are better understood according to their viscosity, gel-forming capacity, fermentability, water-holding capacity, effects on intestinal transit, and ability to modulate nutrient absorption, bile acid metabolism, gut microbiota composition, and SCFA production [10,21]. The major molecular and physiological mechanisms by which dietary fiber influences gut–microbiome–incretin signaling, metabolic inflammation, vascular function, and selected hypothesis-generating neuroimmune contexts are summarized in Table 1 [8,9].

Soluble fibers include psyllium, glucomannan, pectin, beta-glucan, guar gum, inulin, and some hemicelluloses. These fibers dissolve or disperse in water and may form viscous gels within the gastrointestinal lumen. Viscosity is clinically important because it slows gastric emptying, delays intestinal glucose diffusion, reduces the rate of nutrient absorption, and modifies bile acid and cholesterol handling. These effects contribute to the attenuation of postprandial glycemic excursions, the improvement in insulin dynamics, the reduction in LDL cholesterol, and the enhancement of satiety. However, soluble fibers differ substantially in their degree of viscosity and fermentability. For example, inulin is highly fermentable but minimally viscous, whereas psyllium and glucomannan are strongly viscous gel-forming fibers with more pronounced effects on luminal nutrient diffusion and stool consistency [18,21,52].

Insoluble fibers, including cellulose, lignin, wheat bran, and some hemicelluloses, are generally less fermentable and less viscous. Their primary physiological effects are related to fecal bulking, acceleration of intestinal transit, and improvement in bowel regularity. These properties are important for colonic health and constipation prevention but may exert less direct influence on postprandial glycemia, lipid metabolism, and enteroendocrine signaling than viscous soluble fibers. Therefore, insoluble fiber should not be regarded as less important, but rather as functionally distinct. A healthy dietary pattern requires both soluble and insoluble fibers, because stool volume, transit time, microbial fermentation, bile acid metabolism, and cardiometabolic regulation depend on different fiber characteristics [6,21].

Fermentability represents another major determinant of physiological activity. Fermentable fibers are metabolized by colonic bacteria into SCFAs, primarily acetate, propionate, and butyrate. These metabolites serve as signaling molecules that influence intestinal barrier function, immune regulation, lipid metabolism, glucose homeostasis, enteroendocrine hormone secretion, and systemic inflammation. Butyrate is a major energy source for colonocytes and contributes to mucosal integrity, while propionate and acetate participate in hepatic and peripheral metabolic signaling. Through receptors such as GPR41, GPR43, and GPR109A, SCFAs can stimulate GLP-1 and peptide YY release, suppress inflammatory signaling, and influence appetite and insulin sensitivity. Thus, fermentability links dietary fiber intake to host metabolism through the gut microbiome [10,11,15].

However, fermentability alone does not fully determine clinical benefit. Highly fermentable fibers may increase SCFA production but can also cause bloating, gas, and abdominal discomfort in susceptible individuals, particularly when introduced rapidly or in large doses. In contrast, viscous gel-forming fibers such as psyllium may be only partially fermented yet have robust clinical effects through luminal mechanisms, including delayed glucose absorption, altered bile acid recycling, and improved stool water retention. This distinction is important because the metabolic benefits of dietary fiber cannot be reduced to a single mechanism. Rather, different fibers act through overlapping but non-identical pathways [18,21].

This functional classification provides the rationale for focusing on psyllium and glucomannan in this review. Both are viscous soluble fibers with practical availability and evidence supporting effects on metabolic risk factors, yet they differ in their dominant properties. Psyllium combines gel formation, stool normalization, cholesterol modulation, and glycemic attenuation with favorable tolerability. Glucomannan provides high viscosity and water-holding capacity with potential benefits for satiety, weight control, and lipid metabolism. Their structural, physicochemical, safety, and clinical profiles are therefore discussed separately in the following section, while this section establishes the broader framework that fiber physiology depends on function rather than classification alone [19,20,53].

## 4. Psyllium and Glucomannan: Mechanistic Basis and Clinical Relevance

As the principal representative viscous fibers discussed in this review, psyllium and glucomannan are compared mechanistically and translationally in Table 2. Because psyllium and glucomannan are non-digestible, high-molecular-weight polysaccharides that act predominantly within the gastrointestinal lumen, classical pharmacokinetic concepts such as systemic absorption, tissue distribution, hepatic transformation of an intact parent compound, and renal excretion are not directly applicable in the same way as for small-molecule drugs [18,19,21]. Their biological effects are therefore better described as pharmacodynamic-like luminal and microbiome-mediated actions, including hydration-dependent gel formation, increased luminal viscosity, delayed nutrient diffusion, altered bile acid and cholesterol handling, microbial fermentation, SCFA-mediated host signaling, and fecal excretion of unfermented fractions [10,11,21].

**Figure 2:**
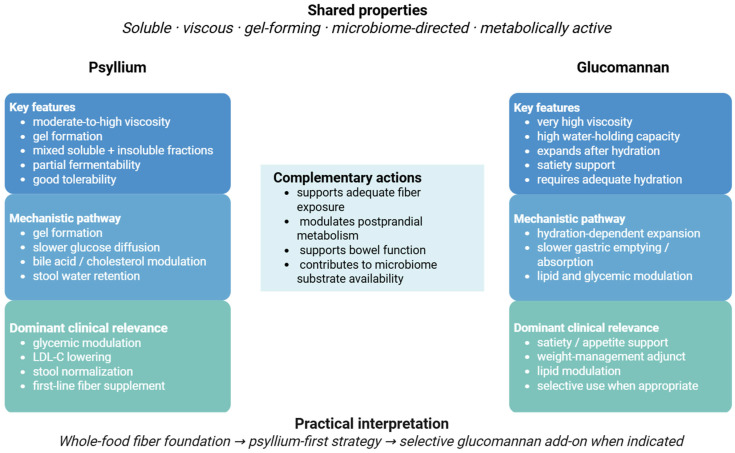
Comparative mechanisms of psyllium and glucomannan as viscous gel-forming fibers. Psyllium and glucomannan share viscous gel-forming properties but differ in dominant clinical and physicochemical profiles. Psyllium supports stool normalization, postprandial glycemic modulation, and LDL-C reduction through hydrated gel formation and partial fermentability. Glucomannan provides higher viscosity and water-holding capacity, which may support satiety and lipid-related outcomes but requires greater attention to hydration and motility status. LDL-C, low-density lipoprotein cholesterol.

The concentration, purity, and fiber content of commercially available psyllium and glucomannan preparations are formulation-dependent and should not be assumed to be identical across powders, capsules, tablets, or mixed products. Accordingly, clinical interpretation should prioritize the amount of functional soluble viscous fiber delivered, hydration conditions, timing with meals or medications, dose titration, gastrointestinal motility, and patient tolerance. Dose–response evidence is stronger for psyllium in relation to LDL cholesterol and glycemic outcomes, whereas glucomannan evidence is more heterogeneous and appears most relevant to viscosity-dependent satiety, lipid modulation, and weight-management support when adequate hydration and appropriate patient selection are ensured [18,20,38,52]. Their shared and complementary mechanisms are further illustrated in Figure 2 [18,19,52].

### 4.1. Psyllium: A Viscous Gel-Forming Fiber with Metabolic and Bowel-Regulating Effects

Psyllium is a soluble, gel-forming fiber derived from the husk of Plantago ovata. It contains both soluble and insoluble fractions and forms a viscous hydrated gel after contact with water. This gel-forming property is central to its physiological effects. Within the gastrointestinal lumen, psyllium increases stool water content, improves stool consistency, slows nutrient diffusion, reduces the rate of glucose absorption, and modifies cholesterol and bile acid metabolism. These actions allow psyllium to influence both bowel function and cardiometabolic risk factors.

The bowel-regulating effect of psyllium differs from that of stimulant laxatives. Rather than directly stimulating intestinal contraction, psyllium absorbs water and forms a soft gel matrix that can normalize stool consistency. In constipation-predominant states, this effect can increase stool bulk and improve ease of passage. In loose stool or diarrhea-prone states, the same water-holding capacity may improve stool form by absorbing excess luminal fluid. This stool-normalizing property is clinically important because many patients with metabolic disease, obesity, or GLP-1 receptor agonist-associated gastrointestinal symptoms experience altered bowel habits [18,54].

Beyond bowel regulation, psyllium has clinically relevant effects on glycemic control. Its viscous gel layer can slow the diffusion of glucose and digestive enzymes, delaying carbohydrate absorption and attenuating postprandial glycemic excursions. This reduction in postprandial glucose exposure may decrease insulin demand and improve insulin dynamics over time. In patients with type 2 diabetes, psyllium supplementation has been associated with reductions in fasting plasma glucose and postprandial glucose responses. In randomized and metabolic studies, psyllium given with meals reduced fasting glucose, postprandial glucose exposure, total cholesterol, LDL cholesterol, and triglycerides, while in some studies, it increased HDL cholesterol [55,56].

Psyllium also exerts lipid-lowering effects, particularly on LDL cholesterol. These effects are related to altered intestinal cholesterol handling, increased bile acid-related cholesterol disposal, and compensatory changes in hepatic cholesterol metabolism. Unlike pharmacological bile acid sequestrants, psyllium does not appear to act simply as a direct bile acid binder in vivo. Rather, it modifies cholesterol absorption and bile acid turnover, contributing to a negative hepatic cholesterol balance and increased LDL clearance. This mechanism is clinically relevant because LDL cholesterol and apoB-containing lipoproteins are central drivers of atherosclerotic cardiovascular risk [18,53].

The tolerability profile of psyllium further supports its clinical relevance. Because psyllium is not as rapidly fermented as some highly fermentable fibers, it may produce less gas and bloating than fibers such as inulin or certain oligosaccharides, particularly when introduced gradually. Adequate hydration remains essential, as insufficient water intake can reduce tolerability and may worsen bloating or constipation. Taken together, psyllium should be considered a metabolically active viscous fiber rather than a simple bulking agent. Its combined effects on stool regulation, postprandial glycemia, LDL cholesterol, satiety, and microbiome-related inflammatory signaling make it a practical candidate for patients with insufficient fiber intake, metabolic syndrome, type 2 diabetes, dyslipidemia, constipation, or reduced dietary volume during weight-loss therapy.

### 4.2. Glucomannan: A High-Viscosity Konjac-Derived Fiber for Satiety, Lipid Metabolism, and Glycemic Modulation

Glucomannan is a high-molecular-weight soluble fiber derived from the root of Amorphophallus konjac. It is characterized by exceptional water-holding capacity and high viscosity. When hydrated, glucomannan expands and forms a viscous matrix within the gastrointestinal tract. This physicochemical behavior provides the basis for its effects on satiety, gastric emptying, nutrient absorption, lipid metabolism, and bowel function. These effects are primarily initiated by luminal physicochemical mechanisms rather than by direct systemic cellular uptake of intact glucomannan; downstream cellular responses may occur secondarily through altered nutrient flux, enteroendocrine L-cell signaling, bile acid handling, microbial fermentation, and SCFA-mediated host signaling [15,19,21].

The high viscosity of glucomannan can delay gastric emptying and slow the interaction between nutrients and the intestinal absorptive surface. This may attenuate postprandial glucose excursions, reduce the rate of lipid absorption, and prolong gastric distension-mediated satiety. These effects are important in patients with obesity, insulin resistance, and metabolic syndrome, where excessive postprandial nutrient flux contributes to hyperinsulinemia, dyslipidemia, oxidative stress, and chronic low-grade inflammation [19,52].

Clinical evidence supports the metabolic relevance of glucomannan. In subjects with insulin resistance syndrome, konjac-mannan supplementation has been associated with improved intermediate glycemic markers and favorable lipid changes, including reductions in total cholesterol, LDL cholesterol, and apoB-related cardiovascular risk markers. Network meta-analytic evidence in type 2 diabetes has also suggested that galactomannan-related viscous fibers may rank favorably for glycemic and lipid outcomes. Glucomannan may further support body weight management because its marked swelling capacity and viscosity can increase fullness, reduce hunger, and lower energy intake when taken before or with meals [20,57,58].

However, the same physicochemical property that makes glucomannan clinically useful also requires caution. Because glucomannan expands substantially after hydration, it should be taken with adequate water and introduced gradually. It may be inappropriate or require careful supervision in patients with dysphagia, esophageal stricture, severe gastroparesis, intestinal obstruction, or marked gastrointestinal motility disorders. This issue requires additional caution during GLP-1 receptor agonist therapy, because these drugs can slow gastric emptying and increase constipation. Therefore, glucomannan should be considered a potentially useful but hydration-dependent and patient-specific fiber intervention [32].

### 4.3. Complementary Rationale for Combined Psyllium-Glucomannan Supplementation

Psyllium and glucomannan share several clinically important properties. Both are soluble, viscous, gel-forming fibers capable of modifying luminal nutrient diffusion, stool hydration, glycemic response, lipid metabolism, and satiety. However, they differ in relative emphasis. Psyllium has a well-established clinical profile for stool normalization, LDL cholesterol reduction, and glycemic attenuation, whereas glucomannan has exceptional water-holding capacity and may be useful for satiety and weight-management goals [18,20,52].

This complementarity provides a rationale for combined supplementation. A combined psyllium-glucomannan model may theoretically address several unmet needs in patients with metabolic disease: inadequate total fiber intake, constipation, postprandial hyperglycemia, dyslipidemia, appetite dysregulation, and low-grade inflammation. In patients receiving GLP-1 receptor agonists, this approach may help address reduced dietary fiber intake caused by appetite suppression and reduced food volume. At the same time, delayed gastric emptying and constipation require careful titration rather than aggressive fiber loading [32,49,50,51].

Combined or sequential use of psyllium and glucomannan should therefore be conceptualized not as a pharmacological treatment or disease-specific therapy, but as a mechanistically informed nutritional strategy for narrowing the gap between habitual and recommended dietary fiber intake. The goal is not to replace whole-food dietary patterns, but to provide a practical bridge for patients who cannot reliably consume sufficient fiber from legumes, vegetables, fruits, whole grains, nuts, and seeds. Mechanistically, a psyllium-glucomannan combination could act through three overlapping domains: a luminal domain involving gel formation and nutrient diffusion; an enteroendocrine domain involving satiety and incretin-related signaling; and a microbiome-immune domain involving SCFA production, gut barrier support, and systemic inflammatory regulation.

Nevertheless, combined fiber supplementation should be individualized. Patients with severe constipation, gastroparesis, reduced fluid intake, swallowing difficulty, or multiple medications require careful dose escalation and counseling. Fiber supplements may also interfere with the absorption of some medications if taken simultaneously; therefore, dose separation is often prudent. In this review, psyllium and glucomannan are used as representative evidence-based viscous fibers, while broader dietary fiber mechanisms are discussed only as contextual pathways that may help explain their luminal, microbial, enteroendocrine, and cardiometabolic relevance.

## 5. Dietary Fiber and Metabolic Syndrome

### 5.1. Viscous and Fermentable Fibers in Metabolic Inflammation and Incretin-Linked Homeostasis

Metabolic syndrome is a clustered cardiometabolic disorder characterized by central obesity, insulin resistance, hyperglycemia, hypertension, elevated triglycerides, and reduced HDL cholesterol. Although these features are often considered as separate clinical risk factors, they share common upstream mechanisms, including excessive postprandial nutrient flux, adipose tissue dysfunction, chronic low-grade inflammation, gut barrier impairment, altered bile acid metabolism, endothelial dysfunction, and dysregulated gut microbiota-derived signaling. Dietary fiber is relevant to metabolic syndrome because it can influence several of these mechanisms simultaneously [59,60].

Fiber effects are determined less by the generic label “fiber” than by physicochemical behavior, particularly viscosity, fermentability, and water-holding capacity. Viscous soluble fibers can form gel-like matrices in the gastrointestinal lumen, slowing nutrient diffusion, delaying glucose absorption, modifying lipid absorption, and altering bile acid recycling. Fermentable fibers provide substrates for colonic microbial metabolism, leading to SCFA production, which influences immune regulation, enteroendocrine hormone secretion, insulin sensitivity, and appetite control. Therefore, dietary fiber should not be viewed as a single nutrient acting through one pathway, but as a group of functional substrates that modify the gut-metabolic axis through overlapping luminal, microbial, endocrine, and inflammatory mechanisms [9,21].

Psyllium and glucomannan fit this metabolic framework because they combine viscosity, gel formation, practical availability, and evidence across multiple metabolic endpoints. Psyllium has been associated with improvements in glucose levels, insulin response, lipid profile, and blood pressure, thereby addressing several components of metabolic risk. Glucomannan, through its high viscosity and water-holding capacity, has been studied in insulin resistance syndrome and has demonstrated favorable effects on glycemic and lipid parameters [52,53].

### 5.2. Glycemic Control and Insulin Resistance

Postprandial hyperglycemia is a major driver of insulin demand, oxidative stress, endothelial dysfunction, and inflammatory activation. In individuals with insulin resistance or type 2 diabetes, rapid glucose absorption after meals contributes to exaggerated glycemic excursions and compensatory hyperinsulinemia. Viscous soluble fibers can attenuate this process by forming a hydrated gel layer that slows the interaction between carbohydrates, digestive enzymes, and the intestinal absorptive surface. This delays monosaccharide absorption and reduces the amplitude of postprandial glucose peaks [61,62].

Psyllium has consistent clinical evidence in this context. By increasing luminal viscosity, psyllium slows glucose diffusion and may reduce postprandial glycemic excursions without directly stimulating insulin secretion. Glucomannan also has glycemic relevance because its high viscosity can delay gastric emptying and slow intestinal glucose absorption. The effects of dietary fiber on glycemic control are not limited to mechanical slowing of absorption. SCFAs produced through microbial fermentation can stimulate enteroendocrine L cells, at least in part through FFAR2/GPR43 and FFAR3/GPR41 signaling, thereby enhancing GLP-1 and peptide YY secretion, improving insulin sensitivity, reducing inflammatory signaling, and influencing hepatic glucose metabolism. Thus, fiber-mediated glycemic regulation involves both immediate luminal effects and longer-term gut microbiome-endocrine interactions [15,40,55,56].

### 5.3. Lipid Metabolism, LDL Cholesterol, and ApoB-Related Risk

Dyslipidemia is a central component of metabolic syndrome and a major contributor to atherosclerotic cardiovascular disease. Elevated LDL cholesterol, triglycerides, and apoB-containing lipoproteins provide the lipid substrate for atherogenesis, while insulin resistance and hepatic steatosis further aggravate lipid abnormalities. ApoB is clinically important because each atherogenic lipoprotein particle, including VLDL remnants, IDL, LDL, and lipoprotein(a), carries one apoB molecule. ApoB therefore reflects the number of circulating atherogenic particles more directly than LDL-C alone and may better capture residual particle-related risk in insulin resistance, hypertriglyceridemia, and metabolic syndrome [63,64]. Viscous soluble fibers can improve lipid metabolism through several mechanisms, including reduced intestinal cholesterol absorption, altered bile acid recycling, increased fecal bile acid excretion, hepatic cholesterol utilization, and compensatory upregulation of LDL receptor-mediated clearance [64,65].

Psyllium has been extensively studied for LDL cholesterol reduction. Its lipid-lowering effect appears to involve modification of cholesterol absorption and bile acid turnover rather than simple direct bile acid binding. Glucomannan may provide complementary lipid-lowering effects through its high viscosity and water-holding capacity. In metabolic syndrome, where elevated triglycerides, small dense LDL particles, insulin resistance, and hepatic lipid dysregulation commonly coexist, viscous fiber interventions may address several lipid and metabolic abnormalities simultaneously. The lipid effects of dietary fiber are clinically important because even modest LDL cholesterol reduction may translate into meaningful long-term cardiovascular risk reduction when sustained over time [20,64].

### 5.4. Body Weight, Satiety, and Appetite Regulation

Obesity and central adiposity are major drivers of metabolic syndrome. Excess adipose tissue, particularly visceral fat, contributes to insulin resistance, chronic low-grade inflammation, altered adipokine secretion, ectopic lipid accumulation, endothelial dysfunction, and increased cardiometabolic risk. Dietary fiber may influence body weight through several mechanisms, including increased gastric distension, delayed gastric emptying, enhanced satiety, reduced energy density of meals, altered bile acid signaling, and SCFA-mediated enteroendocrine hormone release [66,67].

Viscous fibers influence appetite regulation through gel formation, gastric distension, and slower nutrient absorption. Gastric distension may activate vagal afferent satiety signaling, while slower nutrient delivery and SCFA-mediated enteroendocrine L-cell activation may increase GLP-1 and peptide YY release, linking luminal viscosity and microbial fermentation to appetite regulation [15,40,67]. Psyllium and glucomannan can form hydrated gels that increase luminal volume and prolong fullness. Glucomannan, because of its exceptional water-holding capacity, may be especially relevant for satiety and weight management. Psyllium may also reduce appetite by slowing nutrient absorption and stabilizing postprandial glycemic fluctuations. These effects may help reduce compensatory hunger and support dietary adherence during weight management [18,52].

Fiber should not be framed as a stand-alone anti-obesity therapy. Its effect size is likely modest compared with pharmacological weight-loss agents, bariatric surgery, or intensive lifestyle intervention. Its value lies in its ability to improve satiety, bowel function, glycemic stability, lipid metabolism, and microbiome substrate availability while supporting long-term dietary quality. In this respect, fiber supplementation is better framed as a supportive strategy for satiety, bowel function, glycemic stability, lipid modulation, and long-term dietary quality rather than as a rapid weight-loss intervention [31,66,67].

### 5.5. Blood Pressure, Endothelial Function, and Low-Grade Inflammation

Hypertension is another major component of metabolic syndrome and is closely linked to insulin resistance, vascular stiffness, endothelial dysfunction, renal sodium handling, sympathetic activation, and systemic inflammation. Although dietary fiber is not primarily considered an antihypertensive intervention, higher fiber intake has been associated with modest improvements in blood pressure and vascular risk profiles. These effects may be mediated indirectly through weight reduction, improved insulin sensitivity, reduced LDL cholesterol, lower inflammation, enhanced endothelial function, and SCFA-mediated vascular signaling [53,68].

SCFAs provide a mechanistic bridge between dietary fiber and vascular-inflammatory regulation. Butyrate, propionate, and acetate can influence immune cell function, suppress pro-inflammatory cytokine signaling, reinforce intestinal barrier integrity, reduce gut-derived endotoxin translocation, and modulate vascular tone. In metabolic syndrome, where gut barrier dysfunction and low-grade endotoxemia may contribute to systemic inflammation, fiber-mediated SCFA production may help attenuate the inflammatory background that promotes endothelial dysfunction and atherosclerosis [13,16]. At the organ and cellular levels, these effects may involve hepatocyte-mediated cholesterol and bile acid handling, endothelial-cell reduction in oxidative and inflammatory adhesion signaling, immune-cell suppression of excessive cytokine responses, and vascular or renal pathways involved in blood pressure regulation [16,65,69].

Taken together, metabolic syndrome provides a strong rationale for targeted dietary fiber intervention. By acting at the intersection of nutrient absorption, bile acid metabolism, gut microbiota, SCFA production, enteroendocrine signaling, inflammation, and vascular risk, dietary fiber can be conceptualized as a foundational metabolic substrate. This framework supports the transition from viewing fiber as a bowel-regulating nutrient to recognizing it as a clinically relevant component of cardiometabolic risk management [31,52,53].

## 6. Fiber-Derived Microbial Metabolites and the Gut–Vascular Inflammatory Axis

### 6.1. Atherosclerosis as a Metabolic-Inflammatory Vascular Disease

Atherosclerosis is no longer understood simply as passive lipid accumulation within the arterial wall. It is a chronic metabolic-inflammatory vascular disease involving apoB-containing lipoprotein retention, endothelial dysfunction, oxidative stress, immune cell infiltration, foam cell formation, vascular remodeling, and plaque progression. Although LDL cholesterol remains a central causal factor, atherosclerotic risk is also strongly influenced by insulin resistance, postprandial dysmetabolism, hypertension, visceral adiposity, gut-derived inflammatory signals, and systemic low-grade inflammation [24,70].

Dietary fiber is relevant to atherosclerosis because it acts on several upstream determinants of vascular injury. Viscous soluble fibers can reduce LDL cholesterol by altering intestinal cholesterol absorption and bile acid metabolism. Fermentable fibers can increase SCFA production, reinforce intestinal barrier integrity, reduce gut-derived endotoxin translocation, and attenuate systemic inflammation. These mechanisms suggest that fiber may influence atherosclerosis through both lipid-dependent and lipid-independent pathways [16,45].

### 6.2. LDL Cholesterol, Bile Acid Metabolism, and ApoB-Related Risk

LDL cholesterol and apoB-containing lipoproteins are major drivers of atherosclerotic plaque initiation and progression. When circulating apoB particles enter and become retained within the arterial intima, they can undergo oxidative and enzymatic modification, triggering macrophage recruitment, foam cell formation, and chronic vascular inflammation. Therefore, even modest sustained reductions in LDL cholesterol may have meaningful long-term implications for atherosclerotic cardiovascular risk [63,64].

Viscous soluble fibers, especially psyllium and glucomannan, can lower LDL cholesterol through several complementary mechanisms. By forming a viscous gel within the intestinal lumen, these fibers can reduce the efficiency of cholesterol absorption, interfere with micellar lipid transport, and modify enterohepatic bile acid cycling. Increased fecal loss of bile acids or cholesterol-derived compounds can promote hepatic conversion of cholesterol into bile acids, thereby reducing hepatic cholesterol stores and increasing LDL receptor-mediated clearance of circulating LDL particles [20,64,65].

Lipid-lowering by dietary fiber should be viewed as supportive rather than substitutive. In patients with established atherosclerotic cardiovascular disease or high LDL cholesterol, evidence-based pharmacological therapy remains essential. However, fiber-rich diets and targeted viscous fiber supplementation may provide a low-risk adjunctive strategy that improves the overall metabolic environment in which atherosclerosis develops [64].

### 6.3. SCFAs, Gut Barrier Function, and Endothelial Inflammation

Beyond lipid-lowering, dietary fiber influences vascular biology through microbial fermentation and SCFA production. Acetate, propionate, and butyrate are produced when fermentable fibers are metabolized by colonic bacteria. These metabolites act not only as energy substrates but also as signaling molecules that regulate immune function, gut barrier integrity, enteroendocrine hormone secretion, lipid metabolism, and inflammatory responses [10,11].

SCFAs may support vascular homeostasis through several mechanisms. First, they reinforce intestinal barrier integrity, thereby reducing translocation of lipopolysaccharide and other microbial products that contribute to metabolic endotoxemia. Second, they modulate immune cell function by promoting regulatory immune responses and suppressing excessive pro-inflammatory cytokine production. Third, they influence endothelial function, oxidative stress, and vascular remodeling. Fourth, they may affect blood pressure regulation and sympathetic activity through gut-derived signaling pathways [13,16,68].

A compromised intestinal barrier is increasingly recognized as a contributor to systemic metabolic inflammation. Low-fiber diets can reduce microbial diversity, decrease SCFA-producing bacteria, impair mucin production, and weaken tight junction integrity. This may allow increased translocation of bacterial products such as lipopolysaccharide into the circulation. Even low-grade chronic endotoxemia can activate innate immune signaling, promote cytokine release, impair insulin signaling, and contribute to endothelial dysfunction. Dietary fiber may therefore reduce atherosclerotic risk by improving the inflammatory tone of the gut-vascular axis [8,13,24].

### 6.4. Gut-Derived Metabolites: TMAO Versus SCFA Balance

The gut microbiome can generate both protective and potentially harmful metabolites. SCFAs are generally considered beneficial in the context of barrier integrity, immune regulation, and metabolic homeostasis. In contrast, trimethylamine N-oxide (TMAO), produced through microbial metabolism of dietary choline, phosphatidylcholine, and carnitine followed by hepatic oxidation, has been associated with atherosclerosis, thrombosis, and adverse cardiovascular outcomes [46,71,72].

Dietary fiber may influence this metabolite balance by reshaping gut microbial ecology. A fiber-rich diet can promote saccharolytic fermentation, increase SCFA production, and reduce the dominance of microbial pathways associated with proteolytic fermentation and pro-atherogenic metabolite generation. Conversely, low-fiber diets may shift microbial metabolism toward less favorable profiles, particularly when combined with high intake of animal fat, refined carbohydrates, and ultra-processed foods. This concept is clinically relevant because cardiovascular risk may be influenced not only by what substrates are consumed, but also by how the microbiome processes them [3,73,74].

### 6.5. Fiber Type, Microbiome Specificity, and the Emerging Gut-Vessel Axis

Not all fibers exert identical effects on the microbiome or vascular biology. Fiber structure, viscosity, solubility, fermentability, molecular weight, and food matrix all influence microbial utilization and host response. Pectin, beta-glucan, inulin, resistant starch, psyllium, and glucomannan may enrich different microbial taxa and produce different SCFA profiles. Therefore, the cardiovascular effects of fiber cannot be generalized without considering fiber type and host context [11,19,21].

Recent experimental work has highlighted the possibility that specific fibers may modulate atherosclerosis through microbiome-dependent mechanisms, supporting the broader concept of a gut-vessel axis. Although direct human evidence remains limited, this concept is important for future research. Clinical trials of dietary fiber and cardiovascular risk should move beyond traditional endpoints such as LDL cholesterol alone and include microbiome composition, SCFA profiles, inflammatory markers, endothelial function, apoB-related lipid measures, and imaging or biomarker indices of atherosclerotic burden [24,45,75]. Importantly, these gut-vessel mechanisms should be interpreted as fiber-type- and host-context-dependent, and evidence from pectin, inulin, resistant starch, beta-glucan, psyllium, or glucomannan should not be automatically generalized across all viscous or fermentable fibers. These vascular mechanisms provide a transition to the subsequent neuroimmune section, where pain-related phenotypes are discussed only as secondary, hypothesis-generating examples of shared metabolic-inflammatory pathways.

## 7. Dietary Fiber, Metabolic Inflammation, and Hypothesis-Generating Neuroimmune Links

### 7.1. The Gut-Inflammation-Pain Axis

Chronic pain is traditionally classified according to anatomical location or dominant pain mechanism, such as nociceptive, neuropathic, nociplastic, inflammatory, or ischemic pain. However, many chronic pain conditions develop within a broader systemic environment characterized by obesity, insulin resistance, dyslipidemia, endothelial dysfunction, oxidative stress, and low-grade inflammation. These metabolic abnormalities can sensitize peripheral nociceptors, impair microvascular perfusion, alter nerve metabolism, and promote neuroimmune activation. Therefore, in selected patients, chronic pain may be better understood not only as a local tissue disorder but also as a downstream manifestation of systemic metabolic-inflammatory dysfunction [25,76]. These examples are discussed to illustrate shared metabolic-inflammatory mechanisms rather than to propose dietary fiber as a disease-specific treatment for pain or psychiatric disorders.

The gut microbiome is an important regulator of systemic inflammatory tone. Low-fiber diets can reduce microbial diversity, decrease SCFA-producing bacteria, impair mucosal barrier function, and increase translocation of bacterial products such as lipopolysaccharide. This process may promote low-grade endotoxemia, innate immune activation, and increased production of pro-inflammatory cytokines, including IL-6 and TNF-alpha. These cytokines can sensitize nociceptors, influence dorsal horn excitability, and contribute to peripheral and central pain amplification [8,13].

SCFAs provide a mechanistic link between dietary fiber and pain-related inflammation. Butyrate, propionate, and acetate can signal through G protein-coupled receptors such as GPR41, GPR43, and GPR109A, while butyrate also acts as a histone deacetylase inhibitor. Through these mechanisms, SCFAs may suppress NF-kappaB-mediated inflammatory signaling, improve epithelial barrier integrity, and modulate immune cell activity. In experimental models, soluble fiber and butyrate have been associated with reduced intestinal inflammation and attenuation of pro-inflammatory microglial gene expression, suggesting a potential link between fiber-supported microbial metabolism and neuroinflammatory regulation. The proposed, hypothesis-generating links between dietary fiber, metabolic inflammation, pain-related phenotypes, and gut–brain neuroimmune pathways are summarized in Table 3 [10,29,41].

### 7.2. Diabetic Neuropathic Pain and Metabolic Nerve Vulnerability

Diabetic neuropathic pain is one of the most clinically important examples of a metabolically linked pain phenotype. In type 2 diabetes and metabolic syndrome, peripheral nerves are exposed to chronic hyperglycemia, dyslipidemia, insulin resistance, oxidative stress, mitochondrial dysfunction, impaired microvascular perfusion, and inflammatory mediators. These insults can damage small fibers, alter axonal energy metabolism, impair Schwann cell function, and promote peripheral sensitization. Pain, burning, tingling, allodynia, and dysesthesia may therefore reflect not only nerve compression or local injury, but also systemic metabolic nerve vulnerability [26,77].

Several fiber-dependent mechanisms may influence this process indirectly. First, viscous fibers such as psyllium and glucomannan can attenuate postprandial glucose excursions and improve glycemic variability, thereby reducing glucose-related oxidative stress. Second, fiber-mediated improvements in lipid metabolism may reduce lipotoxic injury to peripheral nerves and vascular endothelium. Third, SCFA-mediated immune regulation may reduce systemic inflammatory tone, which is increasingly recognized as a contributor to diabetic neuropathy progression. Fourth, improved gut barrier integrity may reduce metabolic endotoxemia and downstream inflammatory activation [13,14,22].

The available evidence does not yet justify claiming that psyllium or glucomannan directly treats diabetic neuropathic pain. However, the metabolic benefits of these fibers provide a biologically plausible rationale for studying adequate fiber intake as part of a comprehensive metabolic strategy for patients at risk of diabetic neuropathy. Future trials should evaluate whether fiber supplementation can modify neuropathy-related outcomes such as small-fiber function, quantitative sensory testing, neuropathic pain scores, inflammatory biomarkers, and glycemic variability [25,78].

### 7.3. Ischemic Pain, Endothelial Dysfunction, and Microvascular Impairment

Ischemic pain arises when tissue perfusion is insufficient to meet metabolic demand. In the lower extremities, this may manifest as claudication, rest pain, or mixed neurovascular pain in patients with peripheral arterial disease, diabetes, or microvascular dysfunction. Ischemic pain is closely linked to atherosclerosis, endothelial dysfunction, impaired nitric oxide bioavailability, oxidative stress, vascular inflammation, and reduced microcirculatory reserve. These abnormalities are frequently aggravated by insulin resistance, dyslipidemia, hypertension, and chronic low-grade inflammation [24,79].

Dietary fiber may be relevant to ischemic pain phenotypes indirectly by modifying the upstream vascular environment. Psyllium and glucomannan can improve LDL cholesterol and glycemic control, both of which are relevant to atherosclerotic burden and endothelial health. Fermentable fibers can promote SCFA production, improve gut barrier function, and reduce inflammatory signaling that contributes to endothelial injury. By shifting the gut microbiome toward SCFA-producing pathways and away from pro-inflammatory metabolic outputs, fiber-rich diets may help support vascular homeostasis. Nevertheless, dietary fiber is not an acute treatment for ischemic pain, and established vascular disease requires appropriate diagnostic evaluation and evidence-based medical or procedural management [16,64].

### 7.4. Adhesive Capsulitis as a Metabolic-Inflammatory Musculoskeletal Phenotype

Adhesive capsulitis, commonly known as frozen shoulder, has traditionally been described as a local capsular disorder characterized by pain, stiffness, synovial inflammation, capsular fibrosis, and restricted glenohumeral motion. However, its strong association with diabetes, thyroid disease, dyslipidemia, and other metabolic conditions suggests that at least a subset of adhesive capsulitis may represent a systemic metabolic-inflammatory musculoskeletal phenotype [27,80].

Several mechanisms may link metabolic disease to adhesive capsulitis. Chronic hyperglycemia can promote advanced glycation end-product accumulation, collagen cross-linking, oxidative stress, and fibroblast activation. Dyslipidemia and endothelial dysfunction may impair capsular microcirculation and contribute to local inflammatory-fibrotic changes. Low-grade systemic inflammation may further amplify synovial inflammation, pain sensitization, and fibrotic remodeling. Dietary fiber may be relevant to adhesive capsulitis through its effects on metabolic risk rather than direct capsular remodeling. By supporting glycemic regulation, lipid profiles, body weight management, gut barrier integrity, and inflammatory tone, adequate fiber intake may theoretically modify the systemic environment associated with frozen shoulder risk. However, direct interventional studies evaluating fiber supplementation in adhesive capsulitis are lacking [14,81].

### 7.5. Visceral Inflammatory Pain and Bowel Dysmotility

Visceral pain conditions provide another clinically relevant connection between dietary fiber, gut microbiota, and inflammation. Low dietary fiber intake can contribute to altered stool consistency, slowed transit, constipation, dysbiosis, increased intraluminal pressure, and low-grade mucosal inflammation. These changes may aggravate abdominal discomfort, bloating, diverticular symptoms, and bowel dysmotility. In some individuals, chronic intestinal inflammation and altered gut–brain signaling may contribute to visceral hypersensitivity [82,83].

Psyllium has practical value in this context because of its stool-normalizing properties. Unlike stimulant laxatives, psyllium improves stool form through water retention and gel formation, which may benefit both constipation-prone and loose-stool states. In patients receiving GLP-1 receptor agonists, constipation and reduced food volume can worsen bowel symptoms and reduce dietary fiber exposure. Therefore, psyllium-based supplementation may have practical relevance for maintaining bowel regularity while also supporting metabolic health [18,32].

### 7.6. Gut–Brain Axis, Mood-Related Symptoms, and Neuroinflammation

Dietary fiber may also influence neuroimmune pathways beyond pain-related inflammation through the microbiota-gut–brain axis. Although dietary fibers are not digested by human enzymes, fermentable fibers are metabolized by gut bacteria into SCFAs, including acetate, propionate, and butyrate. These metabolites may influence the central nervous system through multiple routes, including systemic immune modulation, vagal signaling, blood–brain barrier regulation, microglial activity, hypothalamic–pituitary–adrenal axis signaling, and neurotransmitter-related pathways [30,84].

Emerging observational evidence suggests that dietary fiber intake may be inversely associated with depressive symptoms and, less consistently, anxiety-related outcomes. However, most human evidence remains observational, and causality cannot be established. Therefore, dietary fiber should not be presented as a treatment for psychiatric disorders. Rather, fiber sufficiency may be considered a supportive nutritional factor that contributes to a less inflammatory and more metabolically stable gut–brain environment [85,86].

Preclinical evidence provides mechanistic support for this concept. Animal studies suggest that the amount and solubility of dietary fiber can alter gene expression in brain regions involved in neuroimmune and mood regulation, including hippocampal microglia and serotonergic neurons in the dorsal raphe nucleus. These effects may be mediated in part by circulating SCFAs and SCFA receptor signaling. This provides a plausible biological link between fiber-supported microbial metabolism, neuroinflammation, serotonin-related pathways, and mood-related phenotypes [29,87].

Nevertheless, this field remains hypothesis-generating. Future clinical studies should evaluate whether psyllium, glucomannan, or broader fiber-rich dietary interventions can modify depressive symptoms, anxiety symptoms, inflammatory biomarkers, SCFA profiles, gut permeability, and microbiome composition in metabolically impaired populations. Such studies would be informative because depression, anxiety, obesity, diabetes, chronic pain, and cardiovascular disease frequently coexist and share overlapping inflammatory and neuroendocrine mechanisms [88,89].

### 7.7. Neuroimmune Context During GLP-1 Receptor Agonist Therapy

GLP-1 receptor agonist therapy provides a relevant but still understudied context for the metabolic and neuroimmune pathways discussed above. Appetite suppression, reduced food volume, delayed gastric emptying, and constipation may reduce fermentable substrate delivery to the colon, potentially influencing SCFA production, bowel function, and systemic inflammatory tone [31,32]. However, whether psyllium, glucomannan, or broader fiber strategies modify pain- or mood-related outcomes during GLP-1 receptor agonist therapy remains unknown. Therefore, GLP-1 receptor agonist-related implications are discussed more fully in Section 9 as a nutritional and bowel-function framework rather than as evidence for direct neuroimmune or analgesic effects.

## 8. Dietary Fiber as an Ecological Substrate for Microbiome-Directed Care

### 8.1. Limitations of a Probiotic-Only Perspective

Probiotics are widely promoted as a strategy for improving gut microbiome composition and gastrointestinal health. In both clinical practice and public discourse, live microbial supplementation is often perceived as the primary method for restoring the gut microbiome. However, this perspective may be incomplete. The persistence, metabolic activity, and clinical relevance of supplemented microorganisms depend on the ecological conditions of the intestinal environment. If the host diet remains deficient in fermentable substrates, the introduced microbes may have limited capacity to persist, function metabolically, or generate bioactive metabolites [90,91].

The gut microbiome should be understood as an ecosystem rather than a simple collection of organisms that can be replaced by supplementation alone. Microbial communities require appropriate substrates, niche stability, mucosal integrity, and host-microbial signaling to maintain functional resilience. A probiotic-only approach may therefore be insufficient when the underlying ecological substrate remains inadequate. In this context, dietary fiber is not merely an adjunct to probiotics, but a foundational determinant of the microbial environment [35,39].

### 8.2. Dietary Fiber as the Ecological Substrate for Resident Microbiota

Dietary fiber acts as the primary nutritional substrate for many beneficial gut microbial communities. When fermentable fiber reaches the colon, it is metabolized by bacteria into SCFAs and other bioactive compounds. These metabolites function as mediators between diet, microbiota, and host physiology. In this way, fiber does not simply feed bacteria; it shapes microbial metabolism and determines the chemical signals delivered from the gut lumen to the host [10,11].

Low-fiber diets can shift microbial communities away from saccharolytic fermentation and toward less favorable metabolic profiles. Reduced fiber availability may decrease SCFA-producing bacteria, impair mucin layer support, weaken epithelial barrier integrity, and promote low-grade inflammatory activation. Conversely, fiber-rich dietary patterns can support microbial diversity, enhance SCFA production, improve stool quality, and reinforce gut barrier function. The ecological role of fiber also explains why microbiome interventions may fail when diet is not addressed. Introducing probiotic organisms into a fiber-depleted intestinal environment is analogous to planting seeds in nutrient-poor soil [8,92].

### 8.3. Prebiotics, Probiotics, Synbiotics, and Postbiotics: Conceptual Distinctions

A clear distinction among prebiotics, probiotics, synbiotics, and postbiotics is necessary for clinical interpretation. Probiotics are live microorganisms that may confer health benefits when administered in adequate amounts. Prebiotics are substrates selectively utilized by host microorganisms, conferring health benefits through changes in microbial composition or activity. Synbiotics combine probiotics and prebiotics, aiming to provide both the organism and the substrate required for function. Postbiotics refer to preparations of inanimate microorganisms or microbial components that may exert biological effects [35,93].

**Figure 3:**
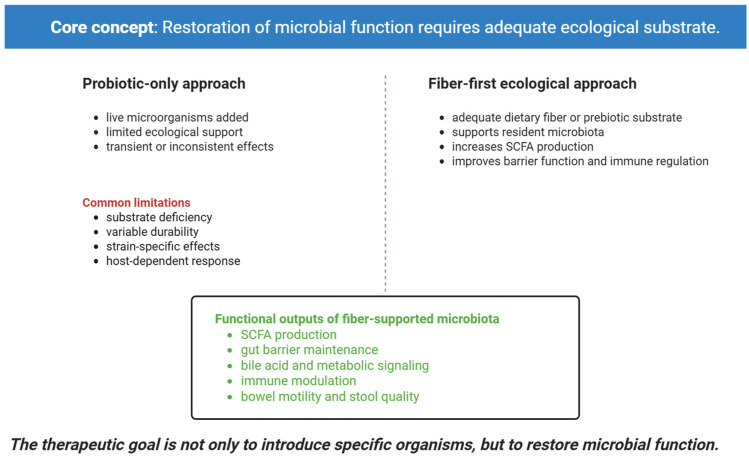
Dietary fiber as an ecological substrate for microbiome-directed interventions. Probiotic and synbiotic strategies may have limited durability when fermentable substrate availability is insufficient. Dietary fiber provides substrate for resident microbial metabolism, short-chain fatty acid production, epithelial barrier support, bile acid signaling, immune modulation, bowel motility, and stool quality. The figure presents a fiber-supported ecological model rather than a probiotic-exclusion model. SCFA, short-chain fatty acid.

Within this framework, dietary fiber is most relevant as a prebiotic or prebiotic-like substrate, depending on fiber type and selectivity. Fermentable fibers such as inulin, resistant starch, pectin, beta-glucan, and certain galactomannans may promote microbial fermentation and SCFA production. Psyllium and glucomannan, although distinct in fermentability and viscosity, may also influence gut microbiota and host metabolism through combined luminal and microbial mechanisms. The clinical target should not be probiotic administration alone, but should include restoration of microbial substrate availability and metabolic function. This substrate-based ecological model is presented in Figure 3 [19,39].

### 8.4. SCFAs as Functional Outputs of Fiber-Supported Microbiota

SCFAs are among the most important functional outputs of fiber-supported microbial metabolism. Acetate, propionate, and butyrate influence host physiology through several mechanisms. Butyrate serves as an energy source for colonocytes, supports tight junction integrity, suppresses inflammatory signaling, and acts as a histone deacetylase inhibitor. Propionate participates in hepatic metabolism, immune regulation, and vascular signaling. Acetate is the most abundant SCFA and contributes to systemic metabolic communication [10,11].

SCFAs also interact with enteroendocrine signaling. Through receptors such as GPR41, GPR43, and GPR109A, SCFAs can stimulate GLP-1 and peptide YY secretion, thereby linking dietary fiber intake to satiety, glycemic regulation, and gut–brain communication. This pathway is relevant during GLP-1 receptor agonist therapy because pharmacological incretin therapy and fiber-derived endogenous incretin signaling may converge on overlapping physiological networks [15,40].

### 8.5. Fiber, Bowel Motility, and Colonic Epithelial Homeostasis

Dietary fiber also has important implications for bowel motility and colonic epithelial homeostasis. Adequate fiber intake supports stool bulk, stool hydration, regular transit, and microbial fermentation. These effects may reduce prolonged contact between the colonic mucosa and potentially harmful luminal contents, improve bowel regularity, and support a healthier colonic microenvironment. In contrast, chronic low-fiber intake may contribute to constipation, dysbiosis, altered bile acid metabolism, low-grade mucosal inflammation, and impaired microbial production of protective metabolites [21,94].

### 8.6. Practical Implications: Fiber as the Foundation, Probiotics as Adjuncts

A practical microbiome-directed strategy should begin with ensuring adequate dietary fiber intake. Whole-food sources, including legumes, vegetables, fruits, whole grains, nuts, and seeds, provide diverse fiber types together with polyphenols, minerals, vitamins, and other bioactive compounds. This diversity is important because different fibers support different microbial taxa and metabolic outputs [95,96].

Fiber supplementation may be useful when whole-food intake is insufficient. Psyllium is practical because it combines stool normalization, viscosity-mediated metabolic effects, and favorable tolerability. Glucomannan may be useful when satiety and high-viscosity effects are desired, although adequate hydration and careful titration are essential. Probiotics may still have a role, but they should be positioned as adjunctive rather than foundational interventions. Their use may be more rational when accompanied by sufficient prebiotic substrate, appropriate diet quality, and attention to bowel motility. In other words, the clinical question should not be fiber or probiotics, but rather how to rebuild the intestinal ecosystem so that beneficial microbial functions can be sustained [18,19,93].

This substrate-based framework leads naturally to the nutritional context of GLP-1 receptor agonist therapy. If pharmacological appetite suppression reduces meal size and plant-food intake, the gut ecosystem may receive less fermentable substrate required for microbial metabolic activity. This issue is discussed in the following section through the lens of nutrient density, fiber exposure, bowel function, and microbiome substrate availability [31,50].

## 9. Dietary Fiber During GLP-1 Receptor Agonist Therapy

### 9.1. A New Nutritional Context Created by GLP-1-Based Pharmacotherapy

GLP-1 receptor agonists and dual GIP/GLP-1 receptor agonists have transformed the management of obesity and type 2 diabetes. These agents improve glycemic control, reduce appetite, delay gastric emptying, promote weight loss, and improve multiple cardiometabolic risk factors. Their clinical impact is substantial, and they are increasingly used not only in patients with diabetes but also in individuals with obesity, metabolic syndrome, and obesity-related complications [97,98,99].

However, the rapid expansion of GLP-1-based pharmacotherapy has created a new nutritional context that requires careful consideration. Because these medications suppress appetite and reduce total food intake, they may unintentionally reduce the intake of fiber-rich foods, including vegetables, legumes, whole grains, fruits, nuts, and seeds. Delayed gastric emptying and gastrointestinal adverse effects such as nausea, bloating, early satiety, and constipation may further discourage consumption of fiber-rich plant foods. As a result, patients receiving GLP-1 RAs may achieve caloric restriction and weight loss while simultaneously reducing fermentable substrate exposure to the gut microbiota [31,50,51].

This issue is clinically important because weight loss alone does not guarantee optimal nutritional quality, bowel function, microbial metabolic activity, or maintenance of metabolic benefits. A patient may lose weight while consuming insufficient dietary fiber, inadequate protein, reduced micronutrients, and limited plant diversity. Therefore, during GLP-1 RA therapy, dietary fiber should be considered not simply as a general healthy diet component, but as a specific nutritional substrate that may require active preservation during pharmacological appetite suppression [100,101,102].

### 9.2. Gastrointestinal Motility, Constipation, and Fiber Exposure

GLP-1 RAs exert part of their therapeutic effect through slowing gastric emptying and altering gastrointestinal motility. These effects contribute to early satiety and reduced energy intake, but they also explain common gastrointestinal symptoms, including nausea, abdominal fullness, bloating, reflux, and constipation. Constipation directly intersects with dietary fiber intake because reduced food volume, reduced fluid intake, delayed transit, and decreased stool bulk can act together to impair bowel regularity [32,49,103].

From a microbiome perspective, gastrointestinal motility influences the timing, quantity, and composition of substrates delivered to the colon. When overall food intake decreases, the absolute amount of fermentable carbohydrate reaching the colon may also decrease. If patients selectively avoid fiber-rich foods because of fullness or bloating, the reduction in microbial substrate may be even more pronounced. This may reduce SCFA production and alter microbial metabolic output. The clinical implication is that GLP-1 RA-associated constipation should not be viewed only as a symptomatic adverse effect. It may also signal a broader change in luminal substrate availability, stool water balance, microbial fermentation, and intestinal transit [31,104,105].

### 9.3. GLP-1 RAs and the Gut Microbiome: Current Evidence and Uncertainty

Emerging evidence suggests that GLP-1 RAs may influence gut microbiome composition, although findings remain heterogeneous and context-dependent. Changes in appetite, caloric intake, gastric emptying, bile acid metabolism, gut transit, and luminal nutrient availability may all affect microbial ecology. Some studies suggest enrichment of microbial taxa associated with metabolic health, such as Akkermansia muciniphila, whereas others show limited or inconsistent changes in alpha diversity, beta diversity, or specific bacterial groups [106,107,108].

This heterogeneity should not be surprising. The gut microbiome response to GLP-1 RA therapy is likely influenced by baseline microbiome composition, diet quality, fiber intake, medication type, dose, duration, degree of weight loss, glycemic status, bowel motility, and host genetics. A key limitation of current research is that microbiome changes are often evaluated without adequate attention to dietary fiber intake. If patients reduce total food intake and fiber exposure during GLP-1 RA therapy, observed microbial changes may reflect not only direct pharmacological effects of incretin signaling but also indirect dietary substrate restriction [15,44,109].

### 9.4. Reduced Fermentable Substrate Availability: A Potential Blind Spot

A potentially underappreciated consequence of GLP-1 RA therapy may be reduced fermentable substrate availability. Appetite suppression is desirable for weight loss, but it can make it more difficult for patients to reach the recommended daily fiber intake. In clinical practice, many patients receiving GLP-1 RAs may prioritize small portions of protein or low-calorie foods while unintentionally reducing legumes, whole grains, fruits, and vegetables. This pattern may be especially pronounced in patients who experience early satiety, nausea, or constipation [31,51,110].

**Figure 4:**
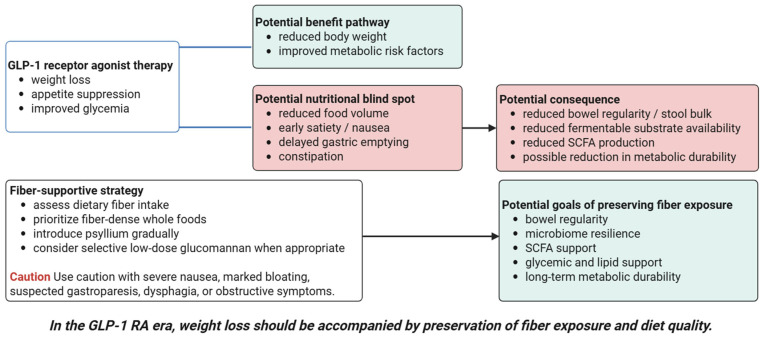
Preserving fiber intake during GLP-1 receptor agonist therapy. GLP-1 receptor agonists improve weight and glycemic outcomes but may reduce meal volume, increase early satiety or nausea, delay gastrointestinal transit, and contribute to constipation. These effects may reduce fiber-rich food intake and fermentable substrate delivery to the colon. A supportive strategy may include a baseline fiber assessment, fiber-dense whole foods, gradual psyllium introduction, and selective low-dose glucomannan use when clinically appropriate and tolerated. Green boxes indicate potential benefits or goals, pink boxes indicate potential nutritional blind spots or consequences, and the red “Caution” label highlights clinical situations in which fiber escalation should be avoided or undertaken carefully. GLP-1 RA, glucagon-like peptide-1 receptor agonist; SCFA, short-chain fatty acid.

Reduced fermentable substrate delivery to the colon may have several consequences. It may decrease SCFA production, reduce ecological support for SCFA-producing bacteria, attenuate endogenous fiber-microbiome-incretin signaling, and worsen constipation by reducing stool bulk and water-holding capacity. This concern does not diminish the therapeutic value of GLP-1 RAs. Rather, it highlights the need for nutritional strategies that preserve fermentable substrate delivery, bowel function, and microbial metabolic output while patients undergo pharmacologically assisted weight loss. The proposed nutritional blind spot and fiber-supportive strategy during GLP-1 RA therapy are summarized in Figure 4 [10,11,31].

### 9.5. Fiber Supplementation as a Supportive Strategy During GLP-1 RA Therapy

Fiber supplementation during GLP-1 RA therapy may be clinically relevant for several reasons. First, it may help compensate for reduced fiber intake from food when appetite and meal volume decrease. Second, it may support bowel regularity and stool consistency, particularly in patients with constipation. Third, it may preserve fermentable substrate availability for the gut microbiota. Fourth, it may support glycemic control, LDL cholesterol reduction, satiety, and inflammatory signaling [18,31,111].

Psyllium is a practical option in this setting because it is a viscous gel-forming fiber with favorable tolerability, stool-normalizing properties, and evidence for metabolic benefits. It can be introduced gradually and adjusted according to bowel response. Glucomannan may also be relevant because of its high viscosity and satiety-promoting effects. However, it should be used more cautiously in patients with pronounced delayed gastric emptying, dysphagia, severe reflux, gastroparesis, or severe constipation. A practical approach is to introduce fiber gradually rather than aggressively. Patients using GLP-1 RAs often already experience fullness and slowed transit; therefore, sudden high-dose fiber loading may worsen bloating or constipation [18,19,32].

### 9.6. Weight Regain After GLP-1 RA Discontinuation: A Microbiome-Centered Hypothesis

Weight regain after discontinuation of GLP-1-based therapy is a major clinical challenge. Pharmacological appetite suppression can produce substantial weight loss, but when medication is reduced or discontinued, hunger, energy intake, and body weight may rebound. This phenomenon is usually explained by neuroendocrine adaptation, appetite regulation, reduced energy expenditure, and behavioral relapse. However, the gut microbiome may also contribute to weight regain vulnerability [34,112,113].

Obesity is associated with altered microbial ecology, reduced metabolic flexibility, low-grade inflammation, and changes in microbial energy harvest. Even after weight loss, some individuals may not fully normalize microbial composition or function. If GLP-1 RA therapy induces weight loss while dietary fiber intake remains low, the microbiome may not receive sufficient substrate to rebuild SCFA-producing capacity, gut barrier integrity, and anti-inflammatory signaling. This could contribute to metabolic vulnerability when pharmacological appetite suppression is withdrawn [31,44,114].

This hypothesis remains to be tested directly. However, it provides a rationale for integrating fiber supplementation during and after GLP-1 RA therapy. The goal would not be to replace medication or independently prevent weight regain, but to support the ecological and metabolic conditions necessary for durable weight maintenance. Future GLP-1 RA discontinuation studies should include dietary fiber intake, microbiome composition, SCFA levels, stool patterns, appetite measures, inflammatory markers, and weight-regain outcomes [31,34,115].

### 9.7. Integrating Fiber with GLP-1 RA Therapy: A Clinical Framework

A rational clinical framework should combine pharmacological and nutritional strategies rather than treating them as separate approaches. GLP-1 RAs can produce substantial appetite suppression and weight loss, while dietary fiber can support bowel function, microbiome substrate availability, glycemic stability, lipid modulation, and inflammatory regulation. These interventions may therefore be complementary [31,51].

Dietary fiber intake should be assessed before and during GLP-1 RA therapy. Fiber-rich whole foods should remain the foundation whenever tolerated. Psyllium supplementation may be considered when whole-food fiber intake is insufficient or when constipation, LDL cholesterol, or glycemic modulation are clinical priorities. Glucomannan may be considered selectively as a high-viscosity adjunct for satiety and lipid-related goals, but it should be used cautiously in patients with significant delayed gastric emptying, swallowing difficulty, or severe constipation. Fiber should be titrated gradually with adequate water intake, and medication timing should be considered because viscous fibers may interfere with the absorption of some oral medications if taken simultaneously [18,32,50].

This integrated framework is important because GLP-1 RA therapy changes eating behavior. As meal size decreases, nutrient density becomes more important. Fiber quality, protein adequacy, micronutrient sufficiency, hydration, and bowel function should be actively managed to prevent pharmacologically assisted weight loss from becoming nutritionally incomplete [100,101,109].

## 10. Practical Fiber Supplementation Framework

### 10.1. Recommended Daily Fiber Intake and the Fiber Gap

Adequate dietary fiber intake is a foundational component of cardiometabolic and gastrointestinal health. As noted above, current dietary guidance commonly recommends approximately 14 g of total fiber per 1000 kcal, corresponding to approximately 25 g/day for adult women and 38 g/day for adult men. European guidance similarly recommends adult fiber intake in the approximate range of 25–35 g/day, with whole grains, fruits, vegetables, pulses, and nuts emphasized as major sources. Despite these recommendations, many adults consume substantially less fiber than recommended, particularly those eating refined, ultra-processed, low-plant-diversity diets [4,5].

This discrepancy between recommended intake and habitual intake may be described as the fiber gap. The fiber gap is clinically important because insufficient fiber intake may reduce stool bulk, impair bowel regularity, decrease fermentable substrate availability for the gut microbiota, reduce SCFA production, and worsen cardiometabolic risk factors. In individuals with low plant-food intake, obesity, metabolic syndrome, or GLP-1 RA-associated dietary restriction, the fiber gap may have greater consequences for bowel function, fermentable substrate delivery, and cardiometabolic regulation [4,11,31].

Fiber supplementation should therefore be framed as a strategy to close the gap between habitual intake and recommended intake, rather than as a substitute for a fiber-rich diet. Whole-food sources remain the preferred foundation because they provide diverse fiber types together with polyphenols, minerals, vitamins, plant proteins, and other bioactive compounds. However, targeted supplementation may be useful when dietary intake is insufficient, poorly tolerated, or limited by reduced appetite, caloric restriction, gastrointestinal symptoms, or medication-induced changes in gut motility [21,95].

### 10.2. Whole-Food Fiber First, Supplementation When Needed

The first step in a practical fiber strategy should be increasing whole-food fiber intake. Legumes, vegetables, fruits, whole grains, nuts, and seeds provide a mixture of soluble, insoluble, viscous, and fermentable fibers. This diversity is important because different fibers influence stool bulk, intestinal transit, microbial fermentation, bile acid metabolism, glycemic response, and satiety through different mechanisms [21,96].

Nevertheless, whole-food fiber intake may be difficult to achieve in many clinical situations. Patients using GLP-1 receptor agonists may have early satiety, nausea, reduced meal volume, constipation, or aversion to bulky foods. Patients with obesity or metabolic syndrome may follow calorie-restricted diets that inadvertently reduce fiber-rich foods. Older adults may have poor appetite, chewing difficulty, reduced fluid intake, or polypharmacy. Patients with irritable bowel symptoms may avoid high-fiber foods because of bloating or discomfort. In these settings, carefully selected fiber supplements can provide a practical and measurable method for increasing dietary fiber intake. A mechanistically informed practical framework for whole-food fiber prioritization, psyllium-first supplementation, selective glucomannan use, and GLP-1 RA-specific considerations is presented in Table 4 [31,32,94].

### 10.3. Psyllium Supplementation: Practical Dose and Clinical Rationale

Psyllium is one of the most practical viscous fiber supplements because it combines stool-normalizing properties with cardiometabolic effects. From a cardiovascular perspective, regulatory health claims in some jurisdictions recognize soluble fiber from psyllium husk as part of a diet low in saturated fat and cholesterol that may reduce the risk of heart disease. Clinical dosing may vary according to formulation, fiber content, and therapeutic goal, but psyllium is commonly introduced gradually and divided with meals when glycemic or lipid modulation is desired [18,61,64]. In practice, dosing should be based on the labeled amount of soluble psyllium fiber rather than total powder weight, because commercial products may differ in husk content, particle size, flavoring agents, and added ingredients. Where dose-specific effects are discussed, the relevant unit should be the grams of soluble psyllium fiber delivered per day or per meal rather than total product weight; a fixed universal dose is not proposed because formulations, clinical goals, background diet, and tolerability differ.

A reasonable approach is to begin with a small labeled serving once daily, taken with sufficient water, and increase stepwise according to bowel response, tolerance, and the estimated fiber gap. Psyllium may be useful when constipation, low fiber intake, type 2 diabetes, dyslipidemia, metabolic syndrome, or GLP-1 receptor agonist-associated reduction in food volume are present. Psyllium should be separated from certain oral medications when clinically appropriate, because viscous fibers may delay or reduce absorption if taken simultaneously [21,54].

### 10.4. Glucomannan Supplementation: Practical Dose and Clinical Rationale

Glucomannan is a high-viscosity, water-absorbing fiber derived from konjac. Its major clinical rationale is based on its ability to expand after hydration and form a viscous matrix that may support satiety, slow nutrient absorption, and modulate lipid metabolism. In some regulatory contexts, glucomannan has been linked to weight reduction when consumed in divided doses together with an energy-restricted diet and to maintenance of normal blood cholesterol concentrations at sufficient daily intake [19,20,52]. Thus, glucomannan dosing should be interpreted in relation to the amount of purified or functional glucomannan delivered, timing before or with meals, hydration status, and gastrointestinal motility rather than as a universally applicable product-level dose.

Because glucomannan swells substantially after water exposure, safety and administration details are especially important. It should be taken with adequate water and should not be used dry, swallowed without sufficient fluid, or escalated rapidly in patients with slowed gastrointestinal transit. It may be inappropriate for patients with swallowing difficulty, esophageal stricture, severe reflux with dysphagia, known gastrointestinal obstruction, severe gastroparesis, or high risk of impaired transit. A practical approach is to use glucomannan selectively rather than universally. It may be most useful when satiety, weight management, or lipid modulation is a priority and when the patient has adequate fluid intake, preserved swallowing safety, and no major motility concerns [32,52].

### 10.5. A Psyllium-Glucomannan Combination Model

A mechanistically informed supplementation model may combine psyllium and glucomannan to take advantage of their complementary properties. Psyllium provides stool normalization, gel-mediated glucose modulation, LDL cholesterol support, and favorable tolerability. Glucomannan provides high viscosity, water-holding capacity, satiety support, and lipid-related effects. Together, they may address multiple needs in patients with metabolic syndrome, dyslipidemia, type 2 diabetes, obesity, constipation, or reduced fiber intake during GLP-1 receptor agonist therapy [18,31,52].

The purpose of this combined model is not to define a single universal product or fixed dose. Rather, it is to propose a flexible framework. For many patients, psyllium may serve as the foundation because of its tolerability and bowel-regulating effect. Glucomannan may be added selectively when appetite control, satiety, or lipid modulation are important goals and when gastrointestinal tolerance is adequate. This staged approach may reduce adverse gastrointestinal symptoms compared with starting multiple fibers at high doses simultaneously [21,94].

### 10.6. Special Considerations in GLP-1 Receptor Agonist Users

Patients receiving GLP-1 receptor agonists require special attention because of reduced appetite, decreased meal volume, delayed gastric emptying, nausea, and constipation. In these patients, the practical goal is not aggressive fiber loading, but gradual preservation or restoration of dietary fiber intake without worsening gastrointestinal symptoms. A gradual psyllium-first strategy may be reasonable because psyllium can improve stool form while providing metabolic benefits [18,31,32].

Fiber counseling in GLP-1 receptor agonist users should include hydration, slow titration, stool monitoring, and attention to food quality. Patients should be encouraged to maintain small but fiber-dense meals when tolerated, including legumes, vegetables, berries, whole grains, nuts, and seeds. If meal volume is very low, fiber supplementation may help maintain stool bulk and microbial substrate exposure. However, if severe nausea, vomiting, marked bloating, suspected gastroparesis, or obstructive symptoms occur, fiber escalation should be avoided, and medical evaluation is required [32,50,51].

### 10.7. Safety, Tolerability, and Contraindications

Although dietary fiber is generally safe, supplementation requires attention to tolerability and patient selection. Because psyllium and glucomannan act primarily within the gastrointestinal lumen rather than through systemic exposure to intact parent compounds, safety concerns are mainly related to gastrointestinal tolerance, hydration, swallowing safety, motility status, and interference with oral medication absorption [18,19,21]. Common adverse effects include bloating, gas, abdominal fullness, cramping, and changes in stool frequency. These symptoms are more likely when fiber is introduced rapidly, taken in large doses, or consumed with insufficient fluid. Adequate hydration is essential for viscous fibers. Psyllium and glucomannan absorb water and form gels; without sufficient fluid, they may worsen constipation or, rarely, contribute to obstruction risk [21,32,94].

Medication timing should also be considered. Viscous fibers can delay gastric emptying and alter drug absorption. Patients taking medications with narrow therapeutic windows or time-sensitive absorption should avoid simultaneous administration with fiber supplements. Fiber supplementation should also be individualized in patients with inflammatory bowel disease flares, severe irritable bowel syndrome symptoms, postoperative bowel changes, strictures, or severe constipation. In such cases, fiber type, dose, and timing should be adjusted according to symptoms and clinical context [21,94].

Overall, fiber supplementation should be presented as supportive nutritional therapy, not as a replacement for evidence-based pharmacological, vascular, diabetic, psychiatric, or pain-specific treatments. This practical framework establishes the basis for the final discussion of limitations and future research needs [31,51].

## 11. Limitations and Future Directions

Although the biological rationale linking dietary fiber to metabolic, vascular, gastrointestinal, inflammatory, and neuroimmune health is strong, several limitations should be acknowledged. First, the available evidence differs substantially according to outcome domain. The clinical evidence for psyllium and glucomannan is relatively stronger for glycemic control, lipid metabolism, bowel regulation, satiety, and selected cardiometabolic markers. In contrast, the evidence linking dietary fiber supplementation directly to improvement in specific pain-related or mood-related conditions remains limited. Importantly, the evidence is not uniformly positive. Some trials and meta-analyses have reported modest, heterogeneous, or clinically variable effects depending on baseline metabolic status, background diet, fiber dose, formulation, adherence, and outcome definition. This is particularly relevant for weight-related outcomes and glucomannan studies, where effects may depend strongly on hydration, timing before meals, energy restriction, and gastrointestinal tolerance. Therefore, the present review should not be interpreted as claiming that psyllium or glucomannan produces consistent benefits across all individuals or all metabolic endpoints [18,20,66].

This distinction is important for scientific interpretation. Dietary fiber should not be presented as a direct analgesic therapy for diabetic neuropathic pain, ischemic pain, adhesive capsulitis, chronic musculoskeletal pain, depression, or anxiety. Rather, it should be interpreted as a potential upstream modifier of metabolic, inflammatory, and neuroimmune pathways that may contribute to these phenotypes. The current evidence supports mechanistic plausibility and indirect clinical relevance, but not definitive therapeutic efficacy for pain or psychiatric outcomes.

Second, dietary fiber is a heterogeneous category. Fibers differ in solubility, viscosity, fermentability, molecular structure, water-holding capacity, food matrix, and microbial utilization. Therefore, findings from one fiber type cannot be automatically generalized to all fibers. Similarly, psyllium-specific evidence should not be automatically extrapolated to glucomannan, and glucomannan-specific findings should not be assumed to apply to psyllium, because their dominant physicochemical properties, fermentability, tolerability, and clinical evidence bases differ. Psyllium, glucomannan, inulin, beta-glucan, pectin, resistant starch, and insoluble cereal fibers may exert different physiological effects. Future studies should avoid treating fiber as a single intervention and should specify fiber type, dose, formulation, timing, and background diet.

Third, much of the microbiome-related evidence remains associative or mechanistic. Changes in microbial composition do not necessarily imply clinically meaningful changes in microbial function. Taxonomic shifts should therefore be interpreted alongside functional endpoints such as SCFA concentrations, bile acid profiles, gut permeability markers, inflammatory biomarkers, enteroendocrine responses, and clinical outcomes.

Fourth, the interaction between dietary fiber and GLP-1 receptor agonist therapy remains insufficiently studied. Although there is a strong rationale that GLP-1 RA-induced appetite suppression, reduced food intake, delayed gastric emptying, and constipation may reduce fiber exposure, direct prospective trials evaluating fiber supplementation in GLP-1 RA users are still lacking. Therefore, the proposed role of psyllium and glucomannan in this setting should be considered hypothesis-generating.

Fifth, the optimal combination of psyllium and glucomannan has not been established. Although their complementary physicochemical properties support a combined supplementation model, direct head-to-head trials comparing psyllium alone, glucomannan alone, and combined psyllium-glucomannan formulations are limited. The ideal dose, ratio, timing, titration schedule, and patient selection criteria remain uncertain.

Sixth, potential interactions between viscous fiber supplements and oral medications remain clinically important but incompletely characterized across patient groups. Psyllium and glucomannan may delay gastric emptying, alter luminal viscosity, and interfere with the timing or extent of absorption of some oral medications, particularly when taken simultaneously. This limitation is especially relevant in patients receiving drugs with narrow therapeutic windows, time-sensitive absorption, polypharmacy, diabetes medications, lipid-lowering therapy, thyroid hormone replacement, or GLP-1 receptor agonist therapy. Practical recommendations should therefore emphasize medication separation, individualized titration, and monitoring rather than universal dosing [21,32,94].

Future research should move from broad dietary fiber associations toward targeted, mechanistically informed, phenotype-specific trials. In metabolic syndrome and type 2 diabetes, randomized trials should evaluate not only fasting glucose, HbA1c, LDL cholesterol, triglycerides, body weight, and blood pressure, but also postprandial glycemic variability, insulin sensitivity, apoB, inflammatory markers, and hepatic steatosis-related outcomes. In atherosclerosis and vascular health, studies should include endothelial function, arterial stiffness, TMAO, SCFA profiles, bile acid metabolites, gut permeability markers, inflammatory biomarkers, and imaging or biomarker indices of atherosclerotic burden.

Pain-related and neuroimmune phenotypes require especially careful study. In diabetic neuropathy, trials could evaluate whether fiber supplementation modifies metabolic and inflammatory intermediates, glycemic variability, small-fiber function, quantitative sensory testing, neuropathic pain scores, and quality of life. In ischemic pain or peripheral arterial disease-related symptoms, studies could assess walking distance, claudication measures, endothelial function, inflammatory markers, lipid profiles, and microvascular perfusion. In adhesive capsulitis, observational studies could first evaluate associations among fiber intake, metabolic syndrome, dyslipidemia, glycemic control, inflammatory markers, and disease severity. In mood-related outcomes, future studies should examine whether fiber-rich diets or targeted fiber supplementation influence depressive or anxiety symptoms through changes in SCFAs, gut permeability, inflammation, and microbiome function.

GLP-1 receptor agonist therapy provides an important research context. Prospective studies should measure dietary fiber intake before and during GLP-1 RA treatment, rather than assuming that weight loss automatically reflects improved diet quality. Randomized trials should evaluate whether psyllium, glucomannan, or combined viscous fiber supplementation can improve constipation, stool consistency, microbiome function, SCFA production, glycemic control, lipid profile, satiety, and adherence during GLP-1 RA therapy. GLP-1 RA discontinuation or dose-reduction studies should include fiber-related endpoints to determine whether fiber sufficiency contributes to maintenance of weight and glycemic benefits after pharmacological appetite suppression is reduced.

Future research should also test this substrate-based framework directly. Studies should compare probiotic-only, prebiotic fiber-only, and synbiotic approaches under controlled dietary conditions. Outcomes should include microbial engraftment, SCFA production, gut barrier markers, bowel function, metabolic markers, inflammatory biomarkers, and patient-reported symptoms. Such studies would clarify whether the clinical target should be microbial abundance, microbial function, metabolite production, or host response.

Ultimately, personalized fiber prescription may become an important direction. Patients differ in metabolic phenotype, bowel habits, microbiome composition, medication exposure, diet quality, and tolerability. A patient with constipation and elevated LDL cholesterol may be a rational candidate for psyllium-centered supplementation. A patient with obesity and preserved motility may be considered for a glucomannan-containing strategy for satiety support. A patient with severe bloating or irritable bowel symptoms may require slower titration or a different fiber profile. Although such precision nutrition approaches are not yet ready for routine clinical use, they represent a logical future direction.

## 12. Conclusions

Dietary fiber is a chronically under-consumed but biologically active nutritional substrate that influences metabolic, vascular, gastrointestinal, immunological, microbiome-related, and selected neuroimmune pathways. Although fiber has traditionally been discussed primarily in relation to bowel regularity, this narrow view does not reflect its broader physiological role. Viscous and fermentable fibers modulate nutrient absorption, bile acid metabolism, gut microbial ecology, SCFA production, intestinal barrier integrity, enteroendocrine signaling, systemic inflammation, vascular homeostasis, and selected neuroimmune pathways.

Psyllium and glucomannan are clinically relevant viscous soluble fibers with complementary properties. Psyllium provides gel-forming, stool-normalizing, LDL cholesterol-lowering, and glycemic-modulating effects with favorable tolerability. Glucomannan provides high viscosity, marked water-holding capacity, satiety support, and lipid-related metabolic effects, although it requires greater attention to hydration, gastrointestinal motility, swallowing safety, and patient selection. Together, these fibers provide a practical model for targeted supplementation when habitual dietary fiber intake is insufficient.

The evidence supporting dietary fiber is strongest for cardiometabolic and gastrointestinal outcomes, including glycemic control, lipid metabolism, body weight regulation, satiety, constipation, and bowel function. The relationship between dietary fiber and pain-related or mood-related phenotypes should be interpreted more cautiously. Current evidence does not support psyllium, glucomannan, or dietary fiber as direct analgesics or psychiatric therapies. However, metabolic syndrome, type 2 diabetes, dyslipidemia, endothelial dysfunction, ischemia, neuroinflammation, and low-grade systemic inflammation share mechanistic overlap with several chronic pain and neuroimmune states. Therefore, fiber may be considered a potential upstream metabolic and microbiome-directed modifier of the inflammatory environment in which these conditions develop or persist.

GLP-1 receptor agonist therapy further increases the relevance of dietary fiber. Pharmacological appetite suppression, reduced meal volume, delayed gastric emptying, nausea, and constipation may reduce the quantity and diversity of fiber-rich foods consumed by patients receiving GLP-1-based therapies. This may decrease fermentable substrate delivery to the colon, potentially affecting microbial metabolic output, SCFA production, bowel regularity, endogenous enteroendocrine signaling, and the maintenance of metabolic benefits. Maintaining adequate dietary fiber intake during and after GLP-1 receptor agonist therapy may therefore be important for bowel regularity, fermentable substrate delivery, microbial metabolic signaling, and cardiometabolic stability.

A substrate-based framework also provides a rational approach to microbiome-directed care. Probiotics may have value in selected contexts, but their durability may be limited if the ecological substrate required for microbial function is insufficient. Dietary fiber provides the nutritional foundation for resident microbial communities, SCFA production, gut barrier maintenance, bowel motility, and host-microbial signaling. In this sense, microbiome-directed care should not be limited to introducing specific microorganisms but should also consider restoration of microbial substrate availability and function.

In practical terms, fiber supplementation should be framed as a strategy to close the gap between habitual intake and recommended intake, rather than as a replacement for fiber-rich whole foods. Whole grains, legumes, vegetables, fruits, nuts, and seeds should remain the foundation of dietary fiber intake. When dietary intake is inadequate or difficult to achieve, psyllium-centered supplementation, with selective addition of glucomannan when appropriate, may provide a clinically realistic and mechanistically informed strategy. Such an approach may be clinically relevant in patients with metabolic syndrome, type 2 diabetes, dyslipidemia, obesity, constipation, low plant-food intake, or GLP-1 receptor agonist-associated gastrointestinal symptoms.

In conclusion, psyllium and glucomannan are clinically accessible viscous fibers that act through luminal, microbial, enteroendocrine, inflammatory, and vascular mechanisms. Their most established roles remain glycemic modulation, LDL-C reduction, stool regulation, satiety support, and cardiometabolic risk modification. During GLP-1 receptor agonist therapy, preserving adequate fiber intake may be important for bowel function, fermentable substrate delivery, and microbial metabolic signaling. Future trials should determine whether these mechanistic effects translate into improved long-term metabolic maintenance and measurable changes in selected neuroimmune or pain-related endpoints.

## Figures and Tables

**Table 1:** Molecular mechanisms linking dietary fiber to gut–microbiome–incretin and inflammatory signaling.

Mechanistic Domain	Fiber-Related Trigger	Key Molecular or Cellular Mediators	Downstream Biological Effect	Relevance to This Review
Luminal viscosity and nutrient diffusion [18,21]	Viscous gel formation by psyllium, glucomannan, beta-glucan, and related fibers	Increased luminal viscosity; delayed carbohydrate and lipid diffusion; altered nutrient–mucosa interaction	Attenuation of postprandial glycemic excursions; slower lipid absorption; reduced postprandial metabolic stress [23,37]	Explains why viscous fibers may support glycemic modulation and metabolic syndrome management
Bile acid and cholesterol handling	Gel-forming soluble fibers interfering with intestinal cholesterol and bile acid dynamics [18]	Bile acid turnover; hepatic cholesterol utilization; LDL receptor-related clearance pathways	Lower LDL-C and apoB-related lipid burden; reduced atherogenic substrate [20,38]	Links psyllium and glucomannan to dyslipidemia, atherosclerosis, and cardiometabolic risk
Microbial fermentation	Fermentable fibers reaching the colon as microbial substrate [35,39]	Saccharolytic bacteria; acetate, propionate, and butyrate production [10,11]	Increased SCFA availability; support of microbial diversity and metabolic output	Supports the concept that fiber functions as an ecological substrate for microbial metabolic function
SCFA–GPCR signaling	Fiber-derived SCFAs acting on host receptors	GPR41/FFAR3, GPR43/FFAR2, GPR109A; immune cells; enteroendocrine L cells [10,14]	Improved insulin sensitivity; immune regulation; GLP-1 and PYY secretion; appetite and glycemic modulation [15,40]	Provides a molecular link between fiber intake, endogenous incretin signaling, and metabolic homeostasis
Butyrate-mediated epigenetic and anti-inflammatory signaling	Butyrate generated through microbial fermentation	Histone deacetylase inhibition; NF-κB-related inflammatory pathways; regulatory immune responses [12,41]	Reduced pro-inflammatory cytokine signaling; support of epithelial and immune homeostasis [8,42]	Connects fiber-derived metabolites to metabolic inflammation and neuroimmune modulation
Intestinal barrier integrity and endotoxemia	Fiber-supported SCFA production and mucosal maintenance	Tight junction proteins; mucin layer; epithelial energy metabolism; LPS translocation [12,43]	Reduced gut permeability and metabolic endotoxemia; lower innate immune activation [8,13]	Links low-fiber states to systemic inflammation, insulin resistance, vascular dysfunction, and selected inflammatory phenotypes
Enteroendocrine and incretin signaling	SCFA-mediated stimulation of intestinal L cells and altered nutrient delivery [15,40]	GLP-1, PYY, L-cell signaling, gut–brain communication	Satiety signaling; improved glycemic control; modulation of appetite and postprandial metabolism	Provides mechanistic overlap between dietary fiber and GLP-1 receptor agonist therapy [31,44]
Vascular inflammation and endothelial function	Fiber-mediated improvements in lipids, glycemia, SCFA signaling, and endotoxemia [24,45]	Endothelial nitric oxide bioavailability; oxidative stress pathways; inflammatory adhesion signaling	Improved endothelial homeostasis; reduced vascular inflammatory tone; lower atherosclerotic risk environment [46,47]	Supports the gut–vascular axis and its relevance to cardiometabolic vascular risk
Neuroimmune and gut–brain signaling	SCFAs and reduced systemic inflammation influencing neural and immune pathways [29,30]	Microglia; vagal signaling; blood–brain barrier regulation; HPA-axis signaling; serotonergic circuits [29,48]	Potential reduction in neuroinflammatory tone; modulation of pain-related and mood-related phenotypes	Frames pain- and mood-related outcomes as secondary, mechanistically plausible contexts rather than direct treatment indications
GLP-1 RA-associated fiber depletion	Appetite suppression, reduced food volume, delayed gastric emptying, and constipation during GLP-1 RA therapy [32,49]	Reduced fermentable substrate delivery; altered bowel transit; reduced SCFA production; microbiome substrate depletion [31,33]	Impaired stool bulk, bowel regularity, microbial metabolic output, and possibly maintenance of metabolic benefits	Supports the rationale for psyllium-centered and glucomannan-selective supplementation during GLP-1 RA therapy [50,51]

This table summarizes key luminal, microbial, enteroendocrine, vascular, and neuroimmune pathways through which dietary fiber may influence host metabolism. The strongest evidence supports cardiometabolic and gastrointestinal outcomes; neuroimmune and pain-related implications should be interpreted as mechanistically plausible but not yet clinically established. This broad classification is used as a functional framework and should not be interpreted as evidence that all fiber types, or both psyllium and glucomannan, exert identical effects across all pathways. Abbreviations: apoB, apolipoprotein B; FFAR, free fatty acid receptor; GLP-1, glucagon-like peptide-1; GLP-1 RA, glucagon-like peptide-1 receptor agonist; GPR, G protein-coupled receptor; HPA, hypothalamic–pituitary–adrenal; LDL-C, low-density lipoprotein cholesterol; LPS, lipopolysaccharide; NF-κB, nuclear factor kappa B; PYY, peptide YY; SCFA, short-chain fatty acid.

**Table 2:** Comparative mechanistic and translational profile of psyllium and glucomannan.

Domain	Psyllium	Glucomannan	Clinical Implication of Complementarity
Source	Husk of Plantago ovata	Konjac root (Amorphophallus konjac)	Both are practical plant-derived viscous fibers suitable for targeted supplementation.
Dominant property	Hydrated gel formation with stool-normalizing capacity	Very high viscosity and water-holding/swelling capacity	Psyllium may be used as a tolerable foundation; glucomannan may add stronger satiety-related viscosity.
Fermentability	Partially fermented; generally less rapidly fermented than inulin-type fibers	Fermentability varies by formulation; dominant feature is high viscosity	A combination can provide luminal viscosity while avoiding excessive reliance on rapidly fermentable fibers.
Bowel effects	Improves stool water retention and stool form; useful in constipation and loose-stool states	May support stool bulk, but can worsen fullness or obstruction risk if poorly hydrated	Psyllium is generally the safer initial option when bowel regularity is the main goal.
Glycemic effects	Slows glucose diffusion and attenuates postprandial glycemic excursions	Delays gastric emptying and nutrient absorption through high viscosity	Both may support glycemic modulation, especially when taken with or before meals.
Lipid effects	Evidence for LDL cholesterol reduction through cholesterol absorption and bile acid-related pathways	Evidence for total cholesterol, LDL cholesterol, and apoB-related lipid improvement in selected studies	Both support cardiometabolic risk management as adjuncts to diet and standard care.
Satiety and weight	May improve fullness and reduce postprandial fluctuations	Marked swelling capacity may enhance satiety and energy intake reduction	Glucomannan may be selectively useful when satiety support is a priority.
GLP-1 RA context	Useful for constipation, stool form, fiber-gap closure, and metabolic support	Use selectively due to delayed gastric emptying and constipation concerns	A psyllium-first, glucomannan-selective strategy is more practical than aggressive combined loading.
Safety focus	Hydration and medication timing; gradual titration	Hydration, swallowing safety, motility status, obstruction risk	Patient selection and stepwise titration are essential.

This table compares psyllium and glucomannan according to source, dominant physicochemical property, fermentability, bowel effects, glycemic and lipid effects, satiety-related mechanisms, GLP-1 receptor agonist context, and safety considerations. Psyllium is positioned as a tolerable foundational viscous fiber, whereas glucomannan is considered a selective high-viscosity adjunct when hydration, swallowing safety, and gastrointestinal motility are adequate. GLP-1 RA, glucagon-like peptide-1 receptor agonist; apoB, apolipoprotein B.

**Table 3:** Proposed links between dietary fiber, metabolic inflammation, and neuroimmune phenotypes.

Phenotype	Metabolic or Inflammatory Substrate	Fiber-Related Mechanistic Link	Evidence Status	Appropriate Manuscript Framing
Diabetic neuropathic pain	Hyperglycemia, dyslipidemia, mitochondrial dysfunction, oxidative stress, microvascular impairment, neuroinflammation	Viscous fibers attenuate glycemic excursions and lipids; fermentable fibers support SCFA-mediated immune regulation and gut barrier integrity	Indirect clinical rationale; direct pain trials remain limited	Fiber as an upstream metabolic modifier, not a direct neuropathic analgesic
Ischemic and microvascular pain	Atherosclerosis, endothelial dysfunction, reduced nitric oxide bioavailability, vascular inflammation, impaired tissue perfusion	LDL reduction, glycemic improvement, SCFA-mediated anti-inflammatory signaling, reduced endotoxemia	Mechanistic and cardiometabolic evidence; direct ischemic pain evidence limited	Fiber as part of cardiometabolic risk reduction influencing vascular pain substrates
Adhesive capsulitis/frozen shoulder	Diabetes, dyslipidemia, AGE accumulation, collagen cross-linking, capsular inflammation and fibrosis	Improved glycemic and lipid control may reduce systemic metabolic-inflammatory burden	Hypothesis-generating; direct interventional studies lacking	Metabolic-inflammatory musculoskeletal phenotype; avoid claiming direct capsular remodeling
Visceral inflammatory pain and bowel dysmotility	Constipation, dysbiosis, mucosal inflammation, altered transit, visceral hypersensitivity	Stool normalization, SCFA production, barrier support, reduced low-grade inflammation	Stronger bowel-function rationale; disease-specific pain evidence variable	Fiber as bowel-regulating and microbiome-supportive therapy with cautious pain implications
Mood-related symptoms and neuroinflammation	Systemic inflammation, HPA-axis dysregulation, microglial activation, serotonin-related pathways, metabolic comorbidity	SCFAs may influence microglia, blood–brain barrier, vagal signaling, and serotonergic circuits	Observational human evidence and preclinical mechanistic evidence; causality uncertain	Gut–brain axis support; not a psychiatric treatment claim
GLP-1 RA-associated metabolic and bowel-function context	Reduced food volume, constipation, reduced fermentable substrate, altered microbiome function, weight-regain vulnerability	Psyllium and selected glucomannan may support dietary fiber intake, stool regularity, and SCFA-related signaling	Timely but under-studied; requires prospective trials	Supportive nutritional strategy during pharmacological weight loss

This table organizes selected pain-related and neuroimmune phenotypes according to plausible metabolic, vascular, inflammatory, and gut-derived mechanisms. These links are intended to guide future research and should not be interpreted as evidence that psyllium or glucomannan directly treats pain or psychiatric disorders. AGE, advanced glycation end-product; HPA, hypothalamic–pituitary–adrenal; SCFA, short-chain fatty acid; GLP-1 RA, glucagon-like peptide-1 receptor agonist.

**Table 4:** Practical framework for psyllium-centered and selective glucomannan supplementation.

Step or Clinical Context	Practical Strategy	Rationale	Cautions and Monitoring
1. Assess the fiber gap	Estimate habitual fiber intake, stool pattern, plant-food diversity, cardiometabolic goals, and medication profile	Fiber supplementation should close the gap between habitual and recommended intake, not replace whole foods	Assess constipation, bloating, dysphagia, gastroparesis risk, and high-risk medications
2. Whole-food fiber first	Prioritize legumes, vegetables, fruits, whole grains, nuts, and seeds as tolerated	Provides diverse fibers plus polyphenols, micronutrients, minerals, and food-matrix benefits	May be difficult with early satiety, nausea, low appetite, IBS symptoms, or GLP-1 RA therapy
3. Psyllium-first supplementation	Start low, titrate gradually with adequate water; consider dosing with meals for glycemic/lipid goals	Practical foundation for stool normalization, LDL support, glycemic modulation, and tolerability	Separate from critical or time-sensitive oral medications when appropriate; monitor bloating, stool response, and hydration adequacy
4. Glucomannan-selective addition	Add selectively for satiety, weight-management, or lipid-related goals when swallowing safety, motility, and hydration are adequate	High viscosity and water-holding capacity may complement psyllium	Avoid or supervise in dysphagia, esophageal strictures, obstruction, severe gastroparesis, severe constipation, or inadequate fluid intake
5. GLP-1 RA users	Use slow titration; prioritize hydration, stool monitoring, and preservation of small fiber-dense meals	GLP-1 RAs may reduce food volume, fiber exposure, and fermentable substrate delivery	Avoid aggressive fiber loading during severe nausea, vomiting, marked bloating, or suspected gastroparesis
6. Cardiometabolic monitoring	Track LDL-C/non-HDL-C, triglycerides, HbA1c or glucose variability, body weight, satiety, and adherence	Fiber effects are multi-domain and cumulative over time	Do not position fiber as a replacement for indicated lipid, diabetes, vascular, or obesity pharmacotherapy
7. Microbiome/neuroimmune research setting	Include SCFAs, stool frequency, gut symptoms, inflammatory markers, pain scores, and mood-related outcomes when feasible	Functional outcomes may be more informative than taxonomy alone	Interpret pain and mood effects cautiously until direct trials are available

This framework is intended for clinical interpretation and future study design. Fiber supplementation should be individualized according to total dietary fiber intake, hydration, bowel function, swallowing safety, gastrointestinal motility, cardiometabolic goals, and concomitant medications. LDL-C, low-density lipoprotein cholesterol; non-HDL-C, non-high-density lipoprotein cholesterol; HbA1c, glycated hemoglobin; IBS, irritable bowel syndrome; GLP-1 RA, glucagon-like peptide-1 receptor agonist; SCFA, short-chain fatty acid.

## Data Availability

No new data were created or analyzed in this study. Data sharing is not applicable to this article.
